# Supplementary material for: Splicing accuracy varies across human introns, tissues, age and disease
Source: Nat Commun. 2025 Jan 27;16:1068. doi: 10.1038/s41467-024-55607-x (PMC11772838; doi:10.1038/s41467-024-55607-x)
Supplement: Supplementary file 1 — Supplementary Information [file 41467_2024_55607_MOESM1_ESM.pdf]

Splicing accuracy varies across human introns, tissues, age and disease

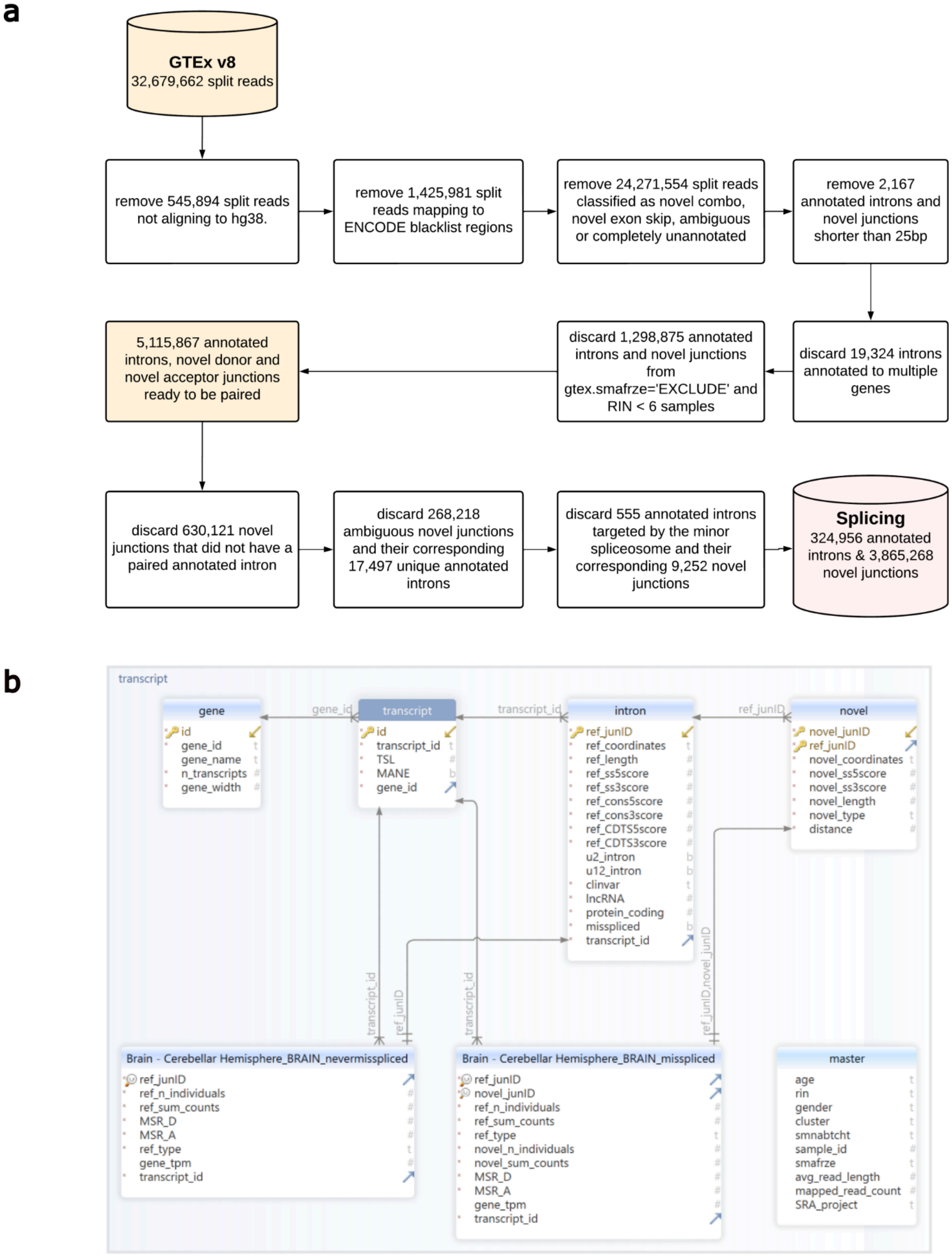

Supplementary Fig. 1. Generation of the Splicing database. a. Overview of the quality-control steps applied to the dataset of exon-exon split reads provided by GTEx v8 to produce the Splicing database. **b.** SQL schema of the Splicing database. Image generated using the software DbSchema 9.2.1 build 230214 (https://dbschema.com/).

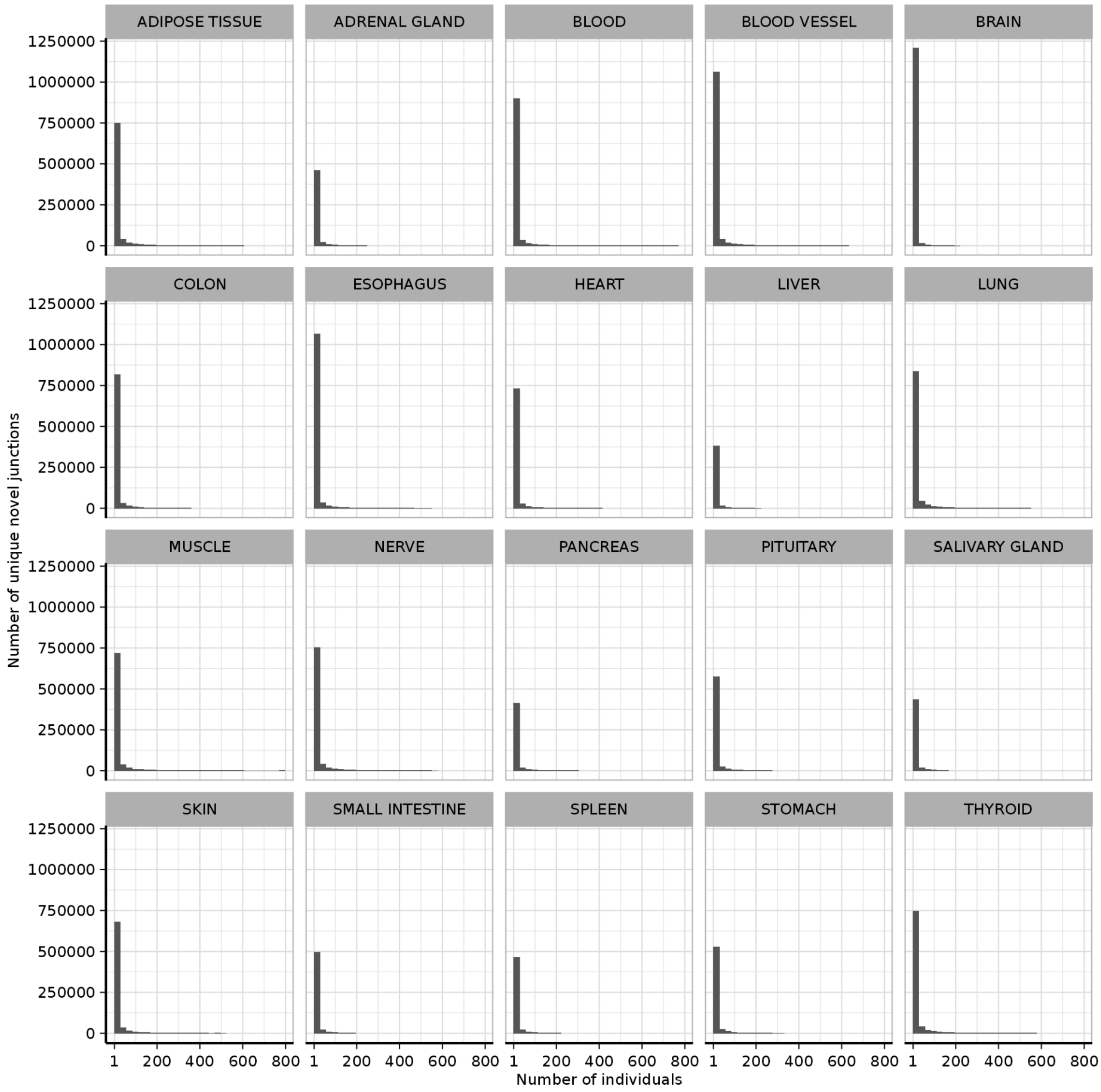

Supplementary Fig. 2. **Novel junction sharing across the sample of each GTEx v8 tissue.** Novel donor and novel acceptor junctions have been collectively considered as novel junctions. The majority of novel junctions are consistently shared across a low number of samples in each GTEx tissue.

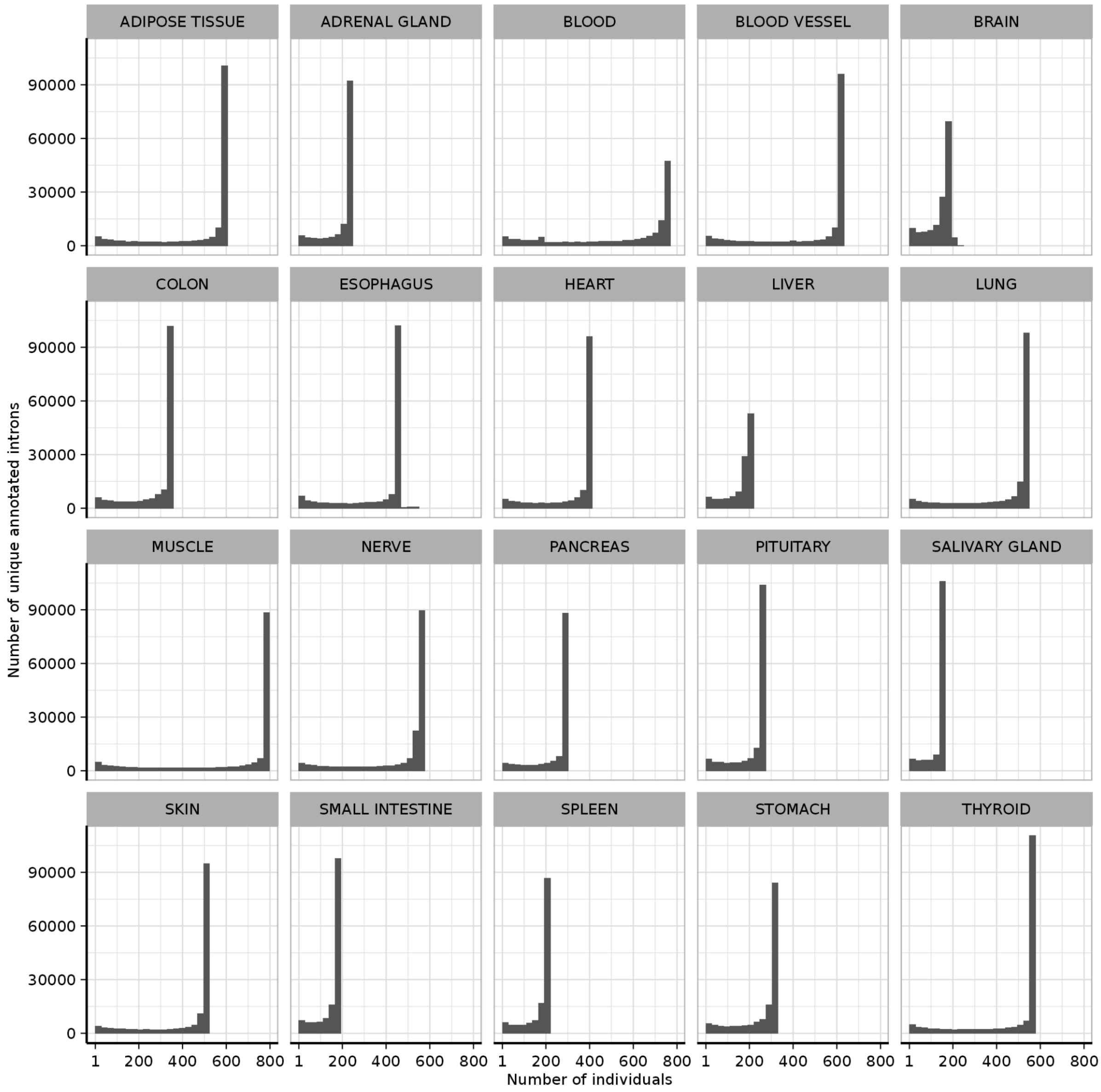

Supplementary Fig. 3. **Annotated intron sharing across the samples of each GTEx v8 tissue.** The majority of annotated introns are consistently shared across a high number of samples in each GTEx tissue.

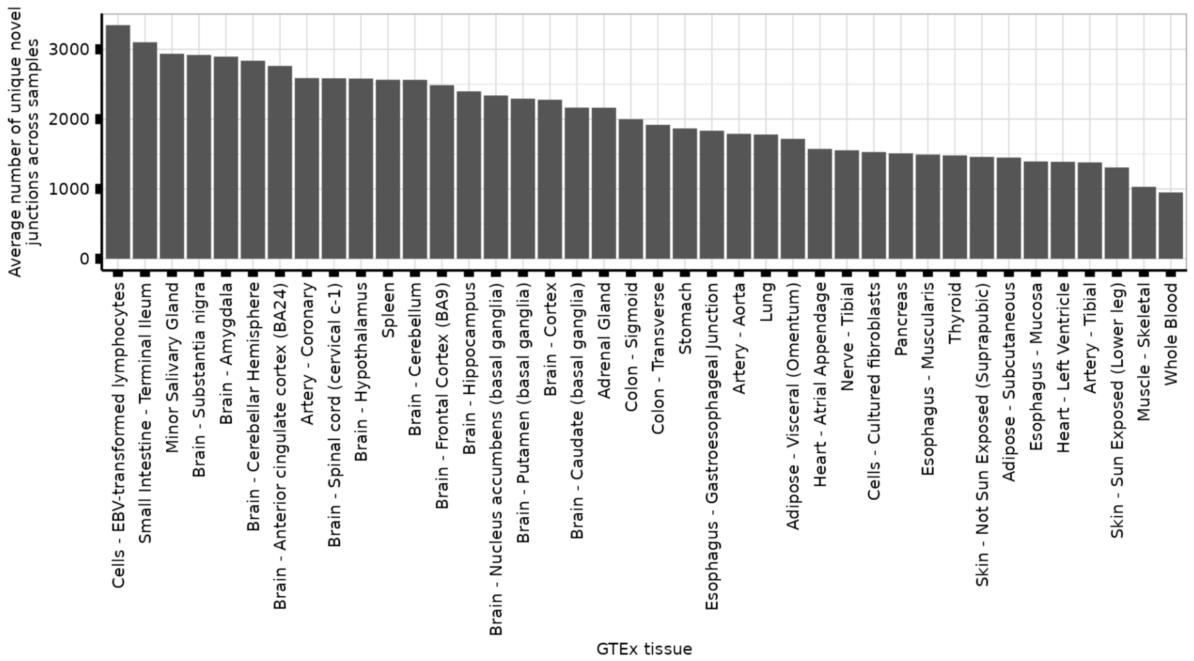

Supplementary Fig. 4. **Average number of novel junctions across the samples of each GTEx tissue.** Novel donor and novel acceptor junctions have been collectively considered as novel junctions.

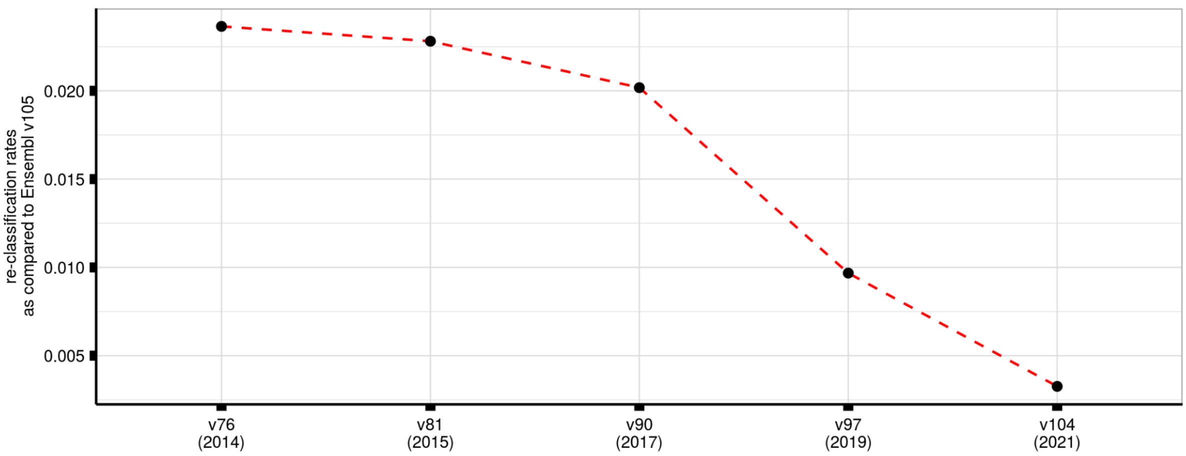

Supplementary Fig. 5. Reclassification rates of novel junctions across Ensembl versions using samples from frontal cortex tissue. Each point represents the percentage of novel junctions in each Ensembl version studied (x-axis) that entered annotation as annotated introns in Ensembl v105 (published in 2021). Re-classification rates were represented between 0 and 1 values, with 0 value representing zero novel junctions entering annotation in v105. Novel donor and novel acceptor splicing events were collectively accounted as novel junctions.

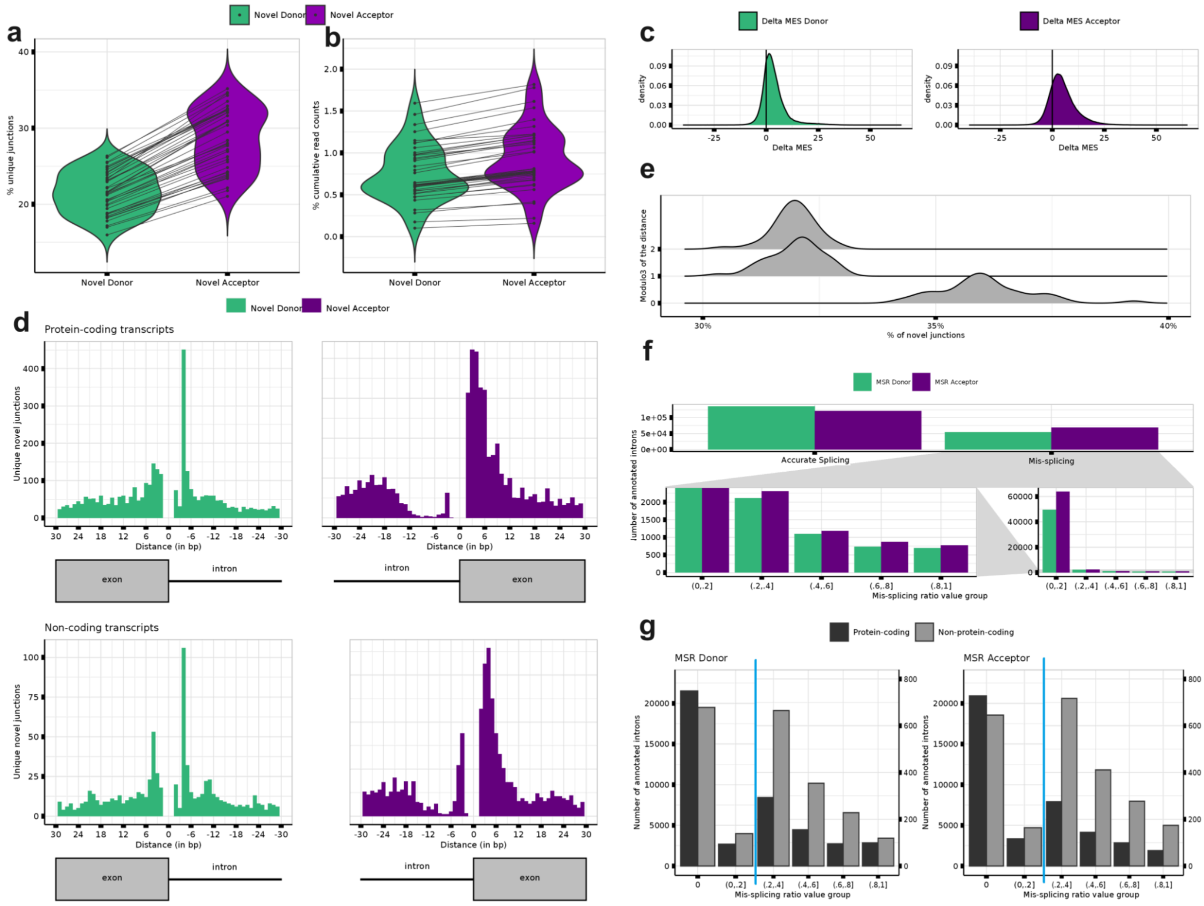

Supplementary Fig. 6. Characterisation of splicing inaccuracies using a minimum of 2 supporting split reads. We re-assessed and characterised splicing noise using novel donor and acceptor junctions supported by at least two independent split reads in at least two of the ~14K GTEx samples studied. a. Percentage of unique novel donor and novel acceptor junctions per GTEx tissue (Ensembl v105). The crossing lines link the percentage of novel donor and novel acceptor junctions found in each body site. **b.** Percentage of cumulative novel donor and acceptor split read counts per GTEx tissue (Ensembl v105). The crossing lines link the percentage of novel donor and acceptor split read count found in each body site. **c.** Delta MaxEntScan (MES) scores between the scores assigned to the 9-bp sequence at the 5'ss (green) and the 23-bp sequence at the 3'ss (purple) of the annotated introns and their novel donor and acceptor pairs, respectively, across all tissues. **d.** Distances between the novel splice site of each novel junction and their annotated pairs from protein-coding and non-protein-coding transcripts in frontal cortex brain tissue. **e.** Modulo3 of the distances between each novel junction and linked annotated intron to a maximum distance of 100 bp within MANE transcripts from all body sites. **f.** MSRs at the 5'ss and 3'ss of the annotated introns in frontal cortex brain tissue samples. Bottom right: MSRs from inaccurately spliced introns across binned values. Bottom left: a zoomed-in view of the bottom right panel, with the y-axis cropped. **g.** MSRs occurring at the 5'ss and 3'ss of the annotated introns located within protein-coding vs non-coding transcripts in samples from frontal cortex brain tissue. The blue vertical line separates the bars represented under the two different y-scales displayed. Right y-scale: a zoomed-in view of the y-axis scale on the left side.

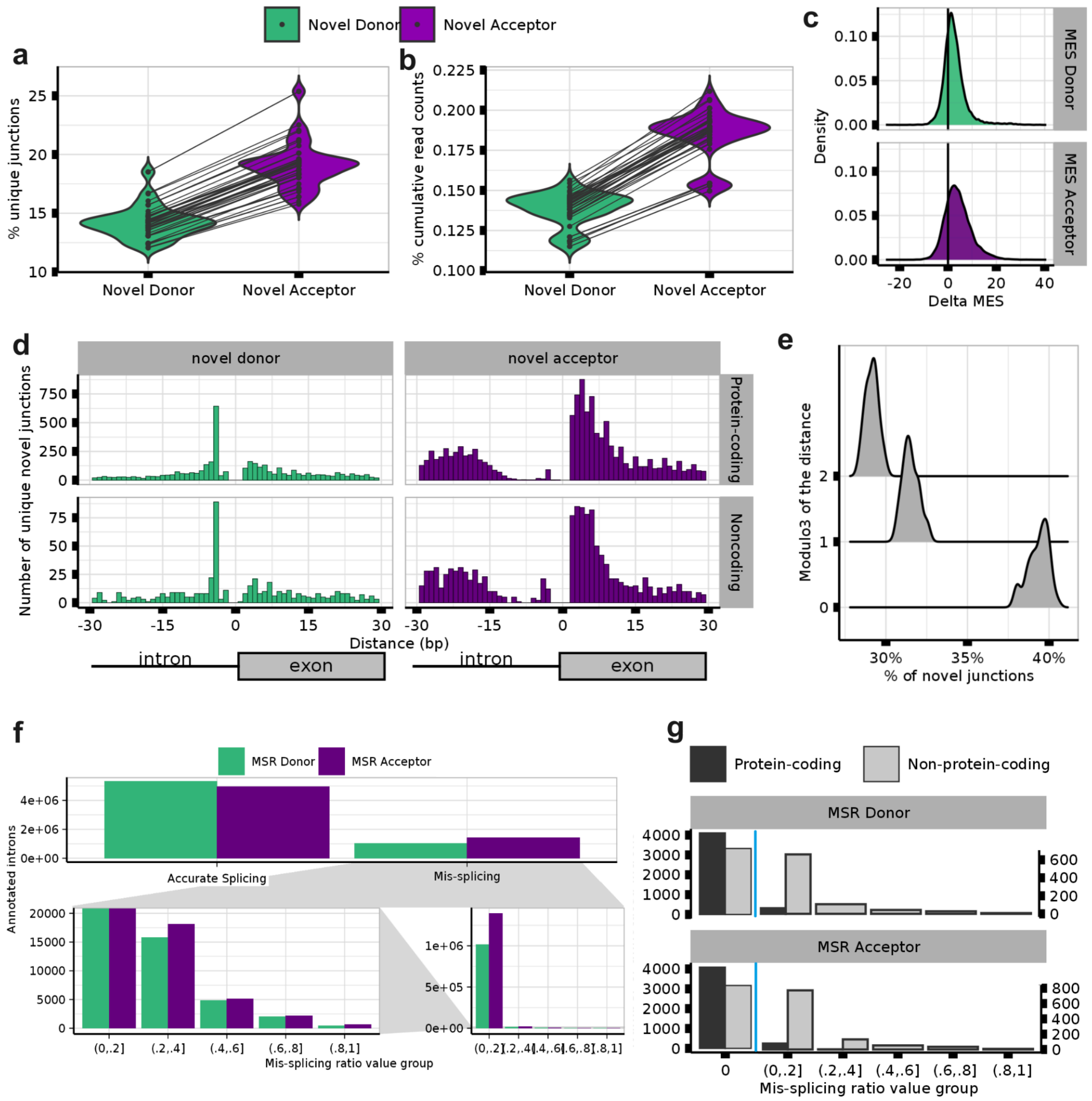

Supplementary Fig. 7. Characterisation of splicing inaccuracies after increasing the anchor length required during read alignment to 8 bp. We re-assessed and characterised splicing noise using 216 shRNA control experiments followed by RNA-sequencing data downloaded from the ENCODE platform. We used a minimum anchor length of 8 bp to call the presence of a split read (RegTools, https://regtools.readthedocs.io/en/latest/). This configuration represented an increase in stringency of 3 bp concerning the anchor size used in the GTEx v8 data (STAR version 2.7.3a, "--outSJfilterOverhangMin = 5", https://gensoft.pasteur.fr/docs/STAR/2.7.3a/STARmanual.pdf). **a.** Proportion of unique annotated introns, novel acceptor and novel donor junctions across the 232 experiments studied. **b.** Proportion of the cumulative number of annotated, novel acceptor and novel donor split reads per experiment. **c.** Delta MaxEntScan (MES) scores between the 5'ss and the 3'ss sequences of the annotated introns and their novel donor (green) and acceptor (purple) pairs. **d.** Distances from novel splice sites to annotated pairs and classified by introns belonging to protein-coding vs non-coding transcripts. **e.** Modulo3 of the distances between novel junctions and their annotated pairs. **f.** MSRs at the 5'ss and 3'ss of the annotated introns. Bottom right: MSRs from inaccurately spliced introns across binned values. Bottom left: a zoomed-in view of the bottom right panel, with the y-axis cropped. **g.** MSRs occurring at the 5'ss and 3'ss of the annotated introns located within protein-coding vs non-coding transcripts.

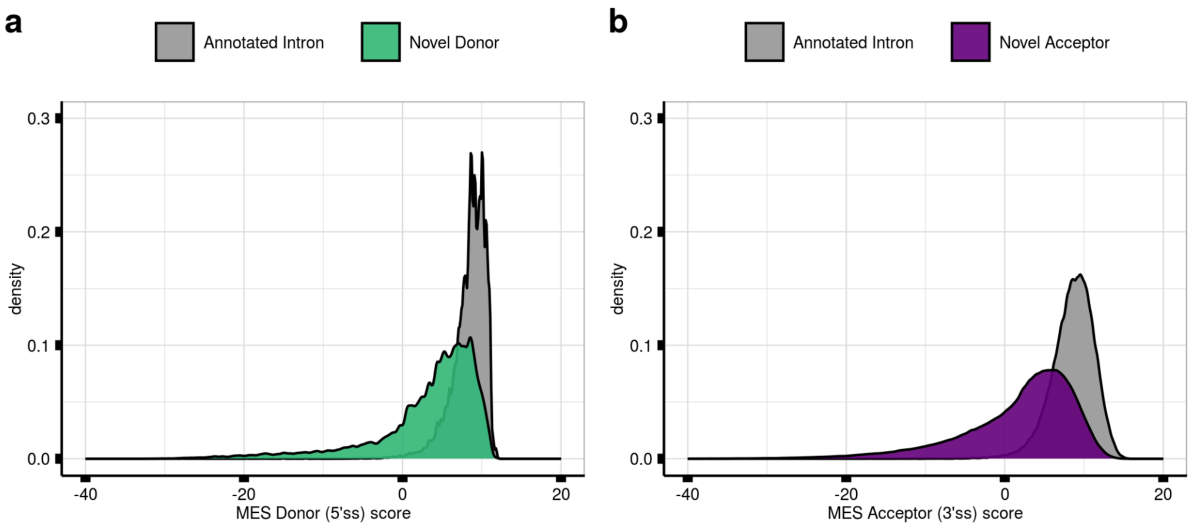

Supplementary Fig. 8. Comparison of the MES scores assigned to the donor and acceptor splice sites of the annotated introns compared to the MES scores assigned to the novel donor and acceptor splice sites of their linked novel junctions. a. MaxEntScan scores assigned to the novel donor splice site (i.e. 5'ss) of all novel donor junctions (n=1,582,593) found across all tissues (in green) and compared with the MES scores assigned to the annotated donor splice site of their linked annotated introns. **b.** MaxEntScan scores assigned to the novel acceptor splice site (i.e. 3'ss) of all novel acceptor junctions (n=2,282,675) found across all tissues (in dark purple) and compared with the MES scores assigned to the annotated acceptor splice site of their linked annotated introns.

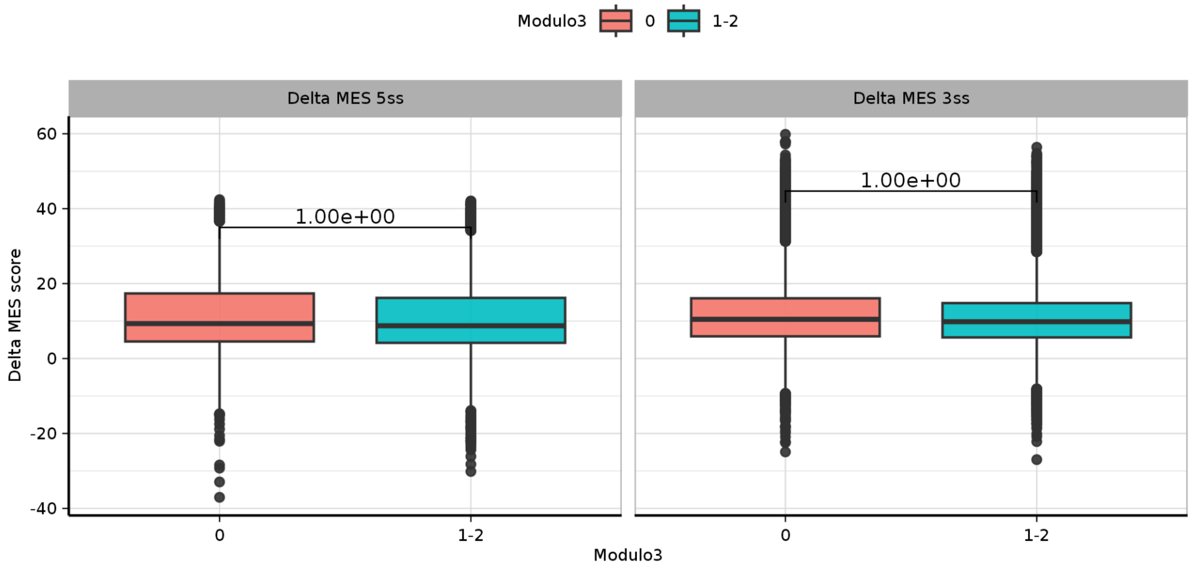

Supplementary Fig. 9. Delta MES at the 5'ss and 3'ss of the novel splice sites located at distances divisible by 3 bp from their annotated pairs (i.e., mod3=0) as compared with those novel junctions not located at distances divisible by three (i.e., mod3=1 & mod3=2). N=243,769 novel splicing events located in close proximity to annotated intron pairs were analysed (maximum distance allowed of 75 bp to their paired annotated sites). One-tailed Wilcoxon Rank-sum test has been performed. Novel junctions located at positions multiple of three bp (mod3=0, n=89,084) did not have a higher degree of motif sequence similarity to their annotated pairs at neither of their two splice sites, represented by lower delta MES, and compared to those novel junctions not located at positions multiple of three (i.e. mod3=1, n=78,748; mod3=2, n=75,937) (one-tailed Wilcoxon Rank-sum test, P=1). Box plots indicate median (middle line), 25th, 75th percentile (box) and 5th and 95th percentile (whiskers) as well as outliers (single points) of the distribution of novel junctions located at distances multiple of 3 bp from annotated pairs (i.e. mod3=0) and those located at distances not multiple of 3 (i.e. mod3=1 and mod3=2).

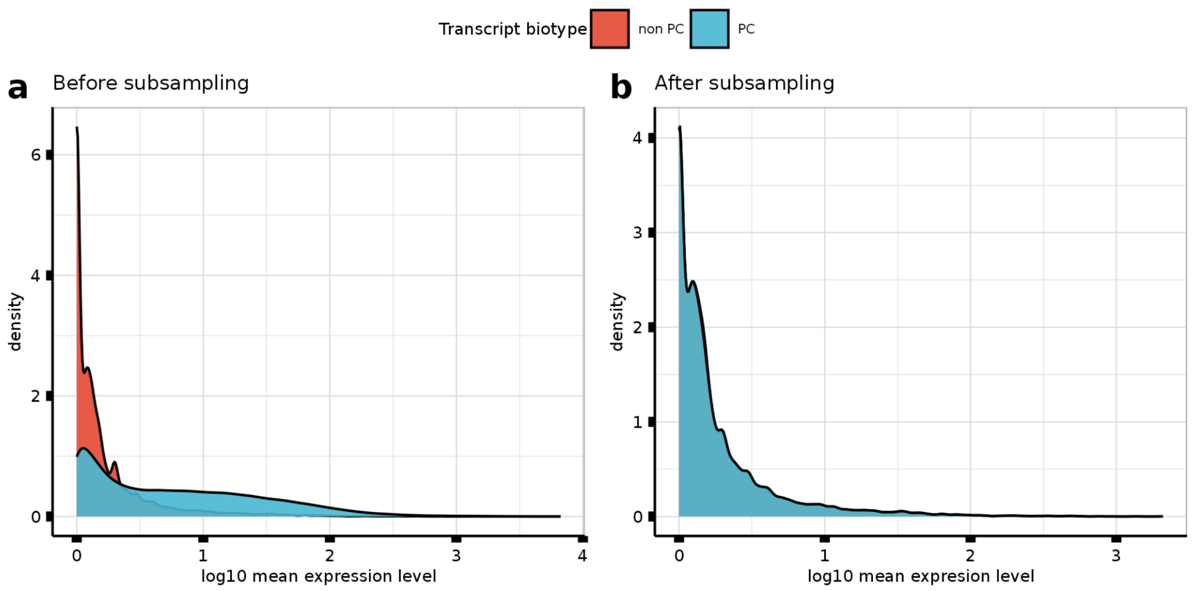

Supplementary Fig. 10. Mean expression levels of the annotated introns from protein-coding and non-coding transcripts in samples from frontal cortex tissue. a. Mean expression levels of the annotated introns from non-coding transcripts (non-PC) (n= 30,474) as compared to the annotated introns from protein-coding transcripts (PC) (n=52,306) before subsampling them to meet similarity in expression levels. **b.** Mean read expression of the annotated introns from non-coding transcripts (non-PC, n= 29,463) as compared to the annotated introns from protein-coding transcripts (PC, n= 29,463) after subsampling them to meet read expression similarity. Only annotated introns indicating a maximum difference of 0.005 in their log10 mean read expression levels between the two data sets (PC and non-PC) were considered, paired and kept for downstream analyses. Mean expression levels were calculated at the intron level by dividing the cumulative number of split reads supporting each annotated intron by the number of samples in which the intron was detected.

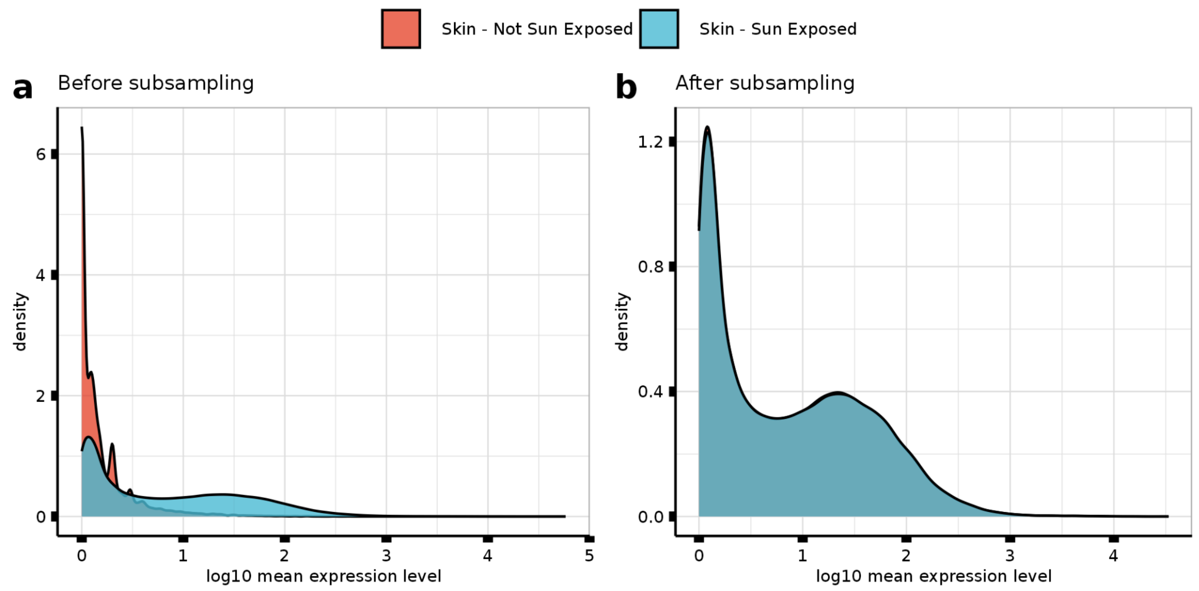

Supplementary Fig. 11. Mean expression levels of the annotated introns from "Skin not-sun-exposed" and "Skin sun-exposed" tissue before and after subsampling them to meet similarity in their mean read expression. a. Mean read expression of the annotated introns from "Skin not-sun-exposed" tissue (n=261,034) versus the annotated introns from "Skin sun-exposed" tissue (n=261,756) before subsampling both distributions to meet for mean read expression similarity. Only annotated introns indicating a maximum difference of 0.005 in their log10 mean read expression between the two tissues were paired and kept for downstream analyses. **b.** Mean read expression of the annotated introns from "Skin not-sun-exposed" tissue (n=245,349) versus the annotated introns from "Skin sun-exposed" tissue (n=245,349) after subsampling and pairing them by mean read expression similarity. Only annotated introns showing a maximum difference of 0.005 in their log10 mean expression between the two tissues were paired and kept for downstream analyses. Mean expression levels were calculated at the intron level by dividing the cumulative number of split reads supporting each annotated intron by the number of samples in which the intron was detected.

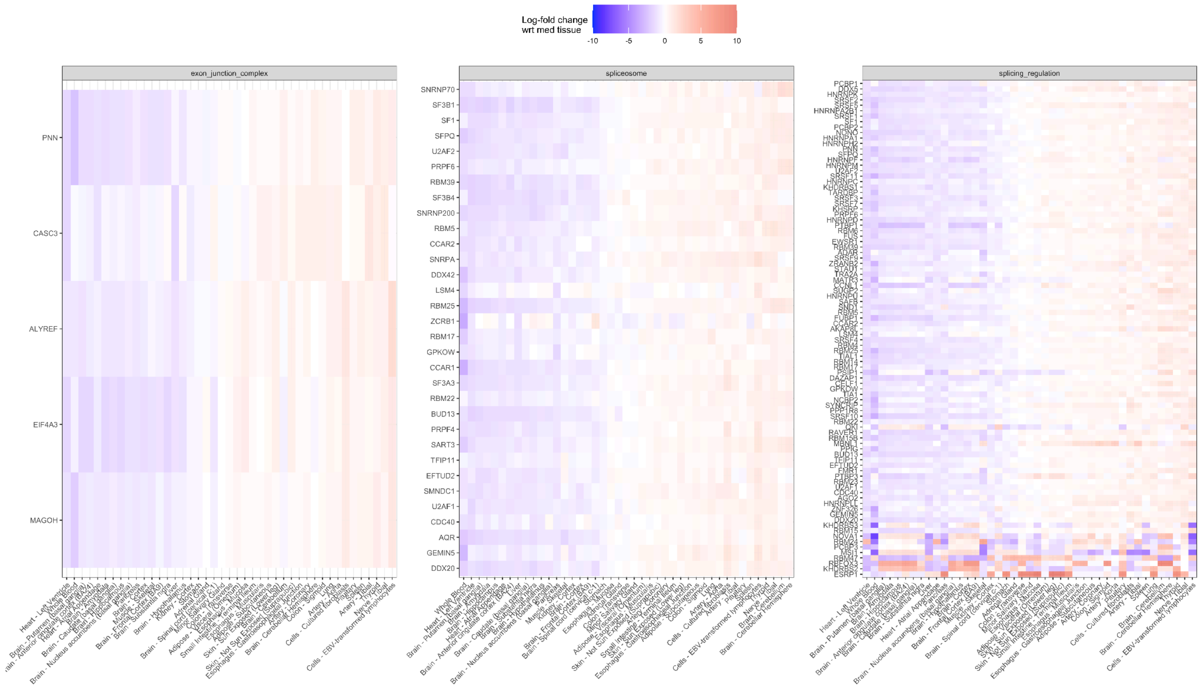

Supplementary Fig. 12. Median expression level of 5 exon-junction complex, 35 spliceosome and 98 splicing-regulator RBPs in 42 GTEx body sites. Log fold-change median expression level per gene across the samples of each one of the 42 GTEx body sites studied. RBPs defined by Van Nostrand et al. 2020.

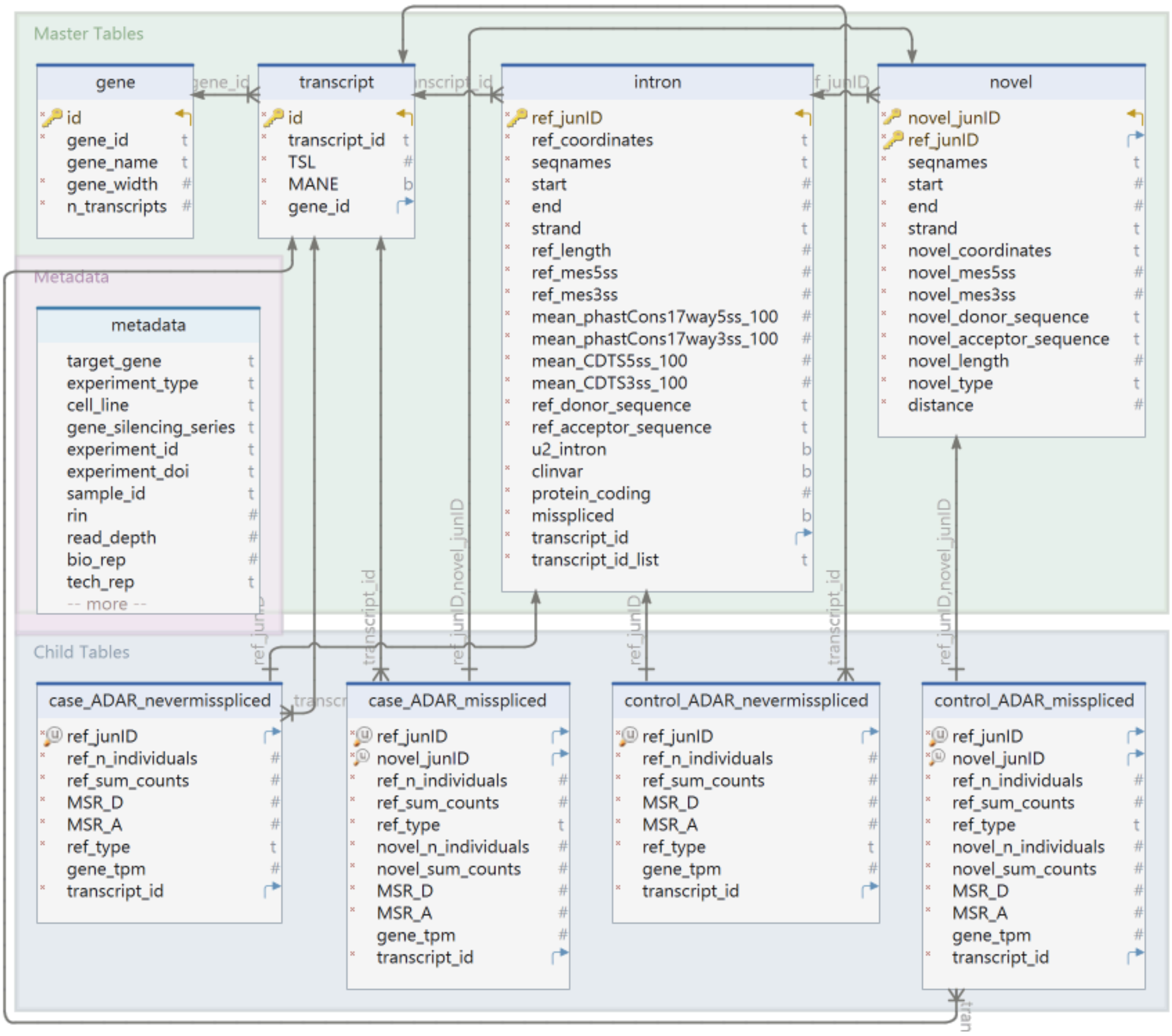

Supplementary Fig. 13. SQL schema of the "ENCODE shRNA" intron database. To facilitate the visualisation of the database structure, only the 4 tables corresponding to the *ADAR* RBP knockdown experiment are shown. These 4 tables store information regarding the accurate and inaccurate splicing activity detected across 4 shRNA experiments targeting *ADAR* and 4 control experiments, separately (*8 samples in total, 4 shRNA knockdown and 4 control experiments). This database was built using publicly available RNA-sequencing data from the ENCODE Gene Silencing Series. Image generated using the software DbSchema 9.2.1 build 230214 (https://dbschema.com/).

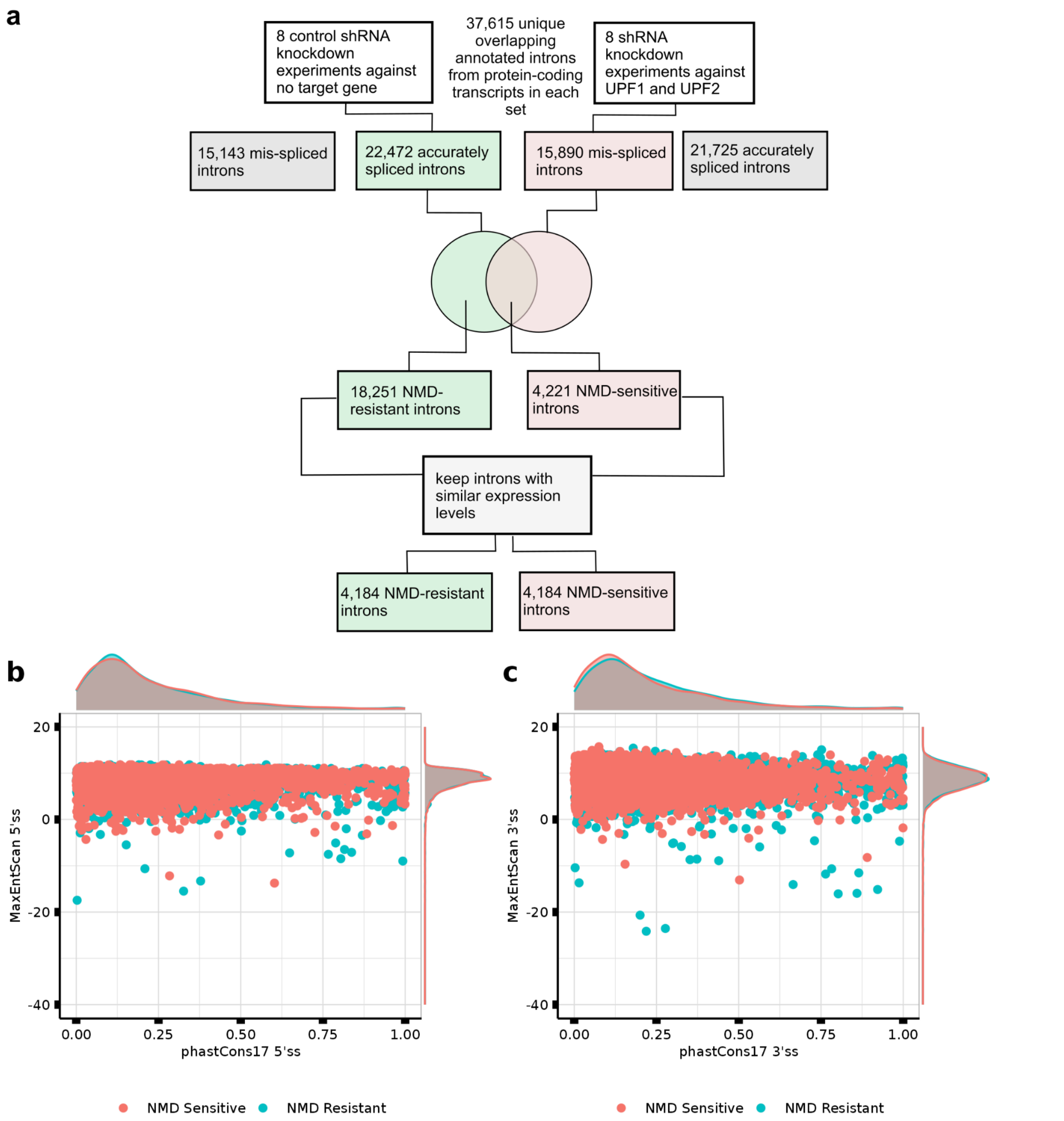

Supplementary Fig. 14. **Detection of NMD-sensitive and NMD-resistant annotated introns and assessment of their level of sequence conservation and splice site strength. a.** We used RNA-sequencing data from the ENCODE Gene Silencing Series corresponding to 8 knockdown experiments against *UPF1* and *UPF2* and 8 controls against no target gene. After data filtering, we considered n=8,368 annotated introns from protein-coding transcripts presenting similar expression levels, from which n=4,184 were classified as *"NMD sensitive"* and n=4,184 were classified as *"NMD resistant"*. **b,c.** Combined scatter plots of phastCons17 (denoting conservation across 17 primates species) and MaxEntScan (denoting motif sequence similarity) of the sequences neighbouring the 5'ss **(b)** and the 3'ss **(c)** of the n=4,184 annotated introns evaluated in each category. *"NMD sensitive"* introns: annotated introns accurately spliced in control experiments with evidence of inaccurate splicing under *UPF1* or *UPF2* knockdown experiments. *"NMD resistant"* introns: annotated introns accurately spliced in control experiments that remained accurately spliced in *UPF1* and *UPF2* knockdown experiments. *"NMD sensitive"* introns are less conserved and weaker at their 3'ss than those from the *"NMD resistant"* category (5'ss phastCons17: paired one-tailed Wilcoxon Rank-sum test, P=0.77; 5'ss MES: paired one-tailed Wilcoxon Rank-sum test, P=0.48) (3'ss phastCons17: paired one-tailed Wilcoxon Rank-sum test, effect-size=0.07, P < 0.001; 3'ss MES: paired one-tailed Wilcoxon Rank-sum test, effect-size=0.03, P = 0.01).

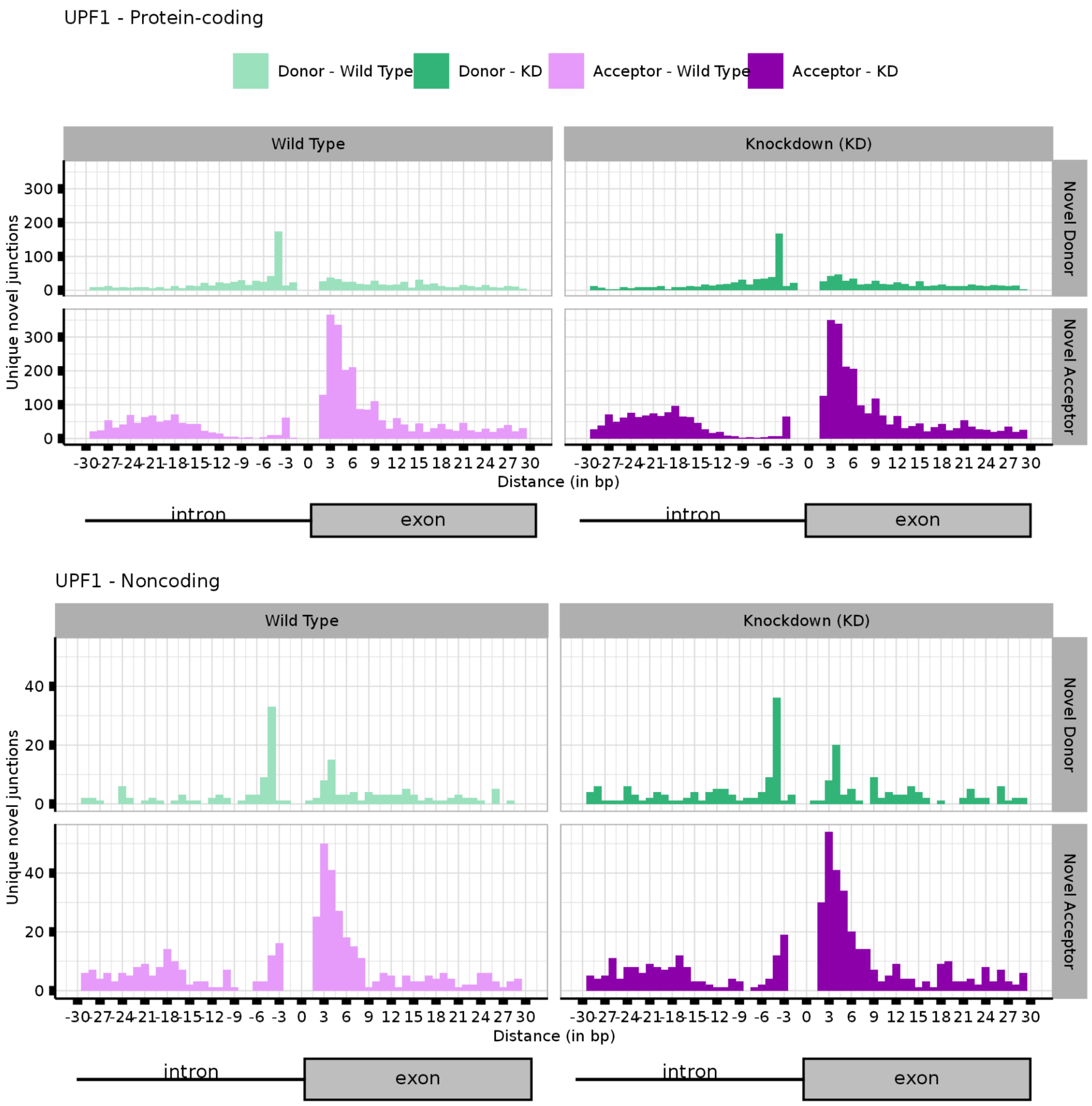

Supplementary Fig. 15. Distances from the novel splice site of each novel donor (in green) and novel acceptor (in purple) splicing events to their annotated pairs in protein-coding vs non-coding transcripts, in samples under knockdown conditions of *UPF1* compared to wild-type samples. The y-axis represents the number of novel donor and acceptor splicing events located at each genomic distance from its annotated pair.

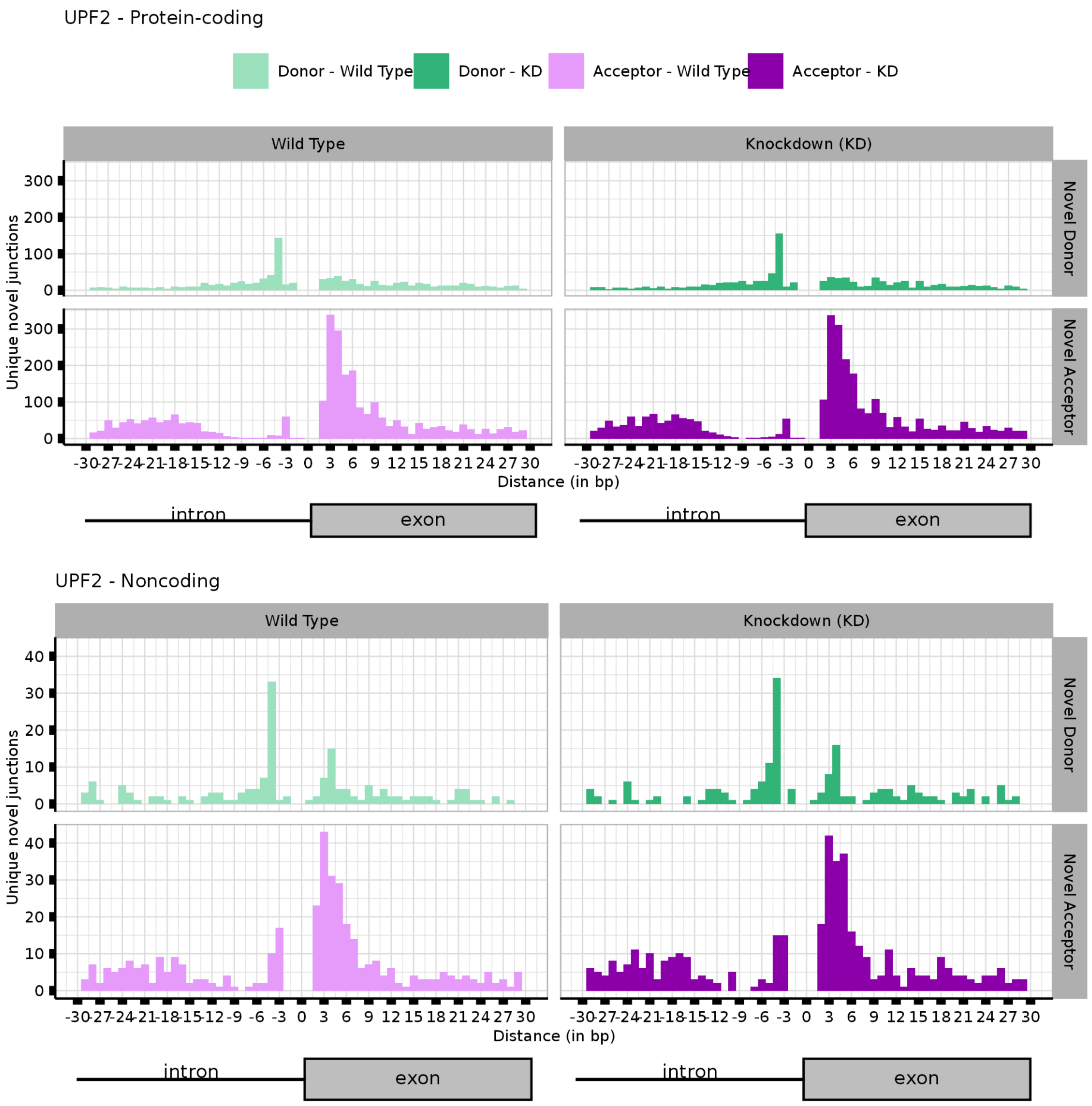

Supplementary Fig. 16. Distances from the novel splice site of each novel donor (in green) and novel acceptor (in purple) splicing events to their annotated pairs in protein-coding vs non-coding transcripts, in samples under knockdown conditions of *UPF2* compared to wild-type samples. The y-axis represents the number of novel donor and acceptor splicing events located at each genomic distance from its annotated pair.

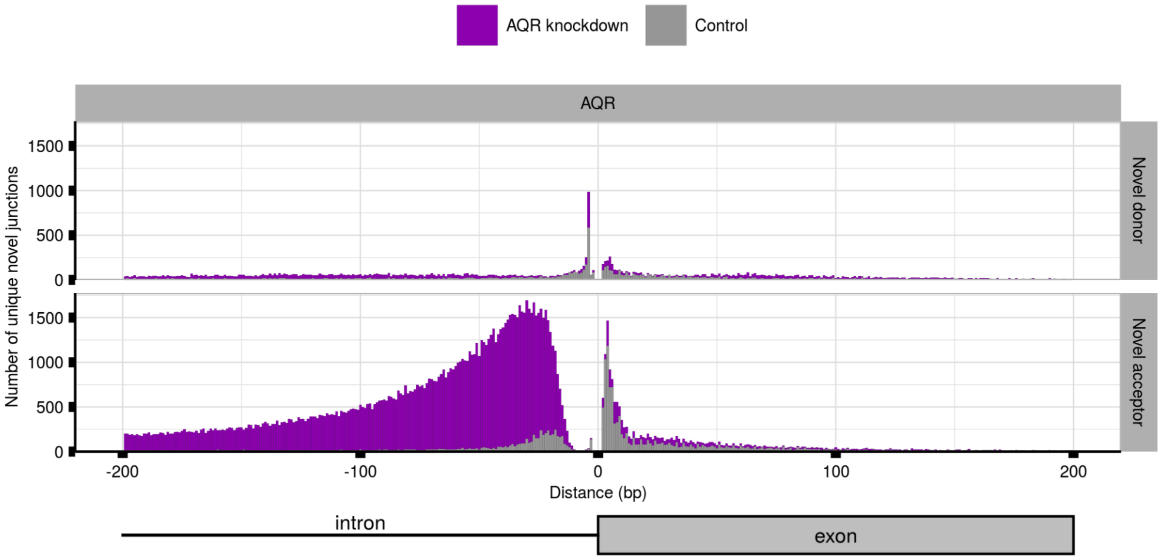

Supplementary Fig. 17. **Distances between the novel splice site of each novel donor and acceptor splicing event and its annotated pair in experiments under shRNA knockdown conditions of *AQR* compared to untreated samples.** Only distances up to 200 bp upstream and downstream each 5’ss and 3’ss annotated splice site have been displayed**.** The y-axis represents the number of novel junctions located at each genomic position from its annotated pair. shRNA knockdown of *AQR* produces considerably high levels of inaccurate splicing at the acceptor site of the annotated introns analysed.

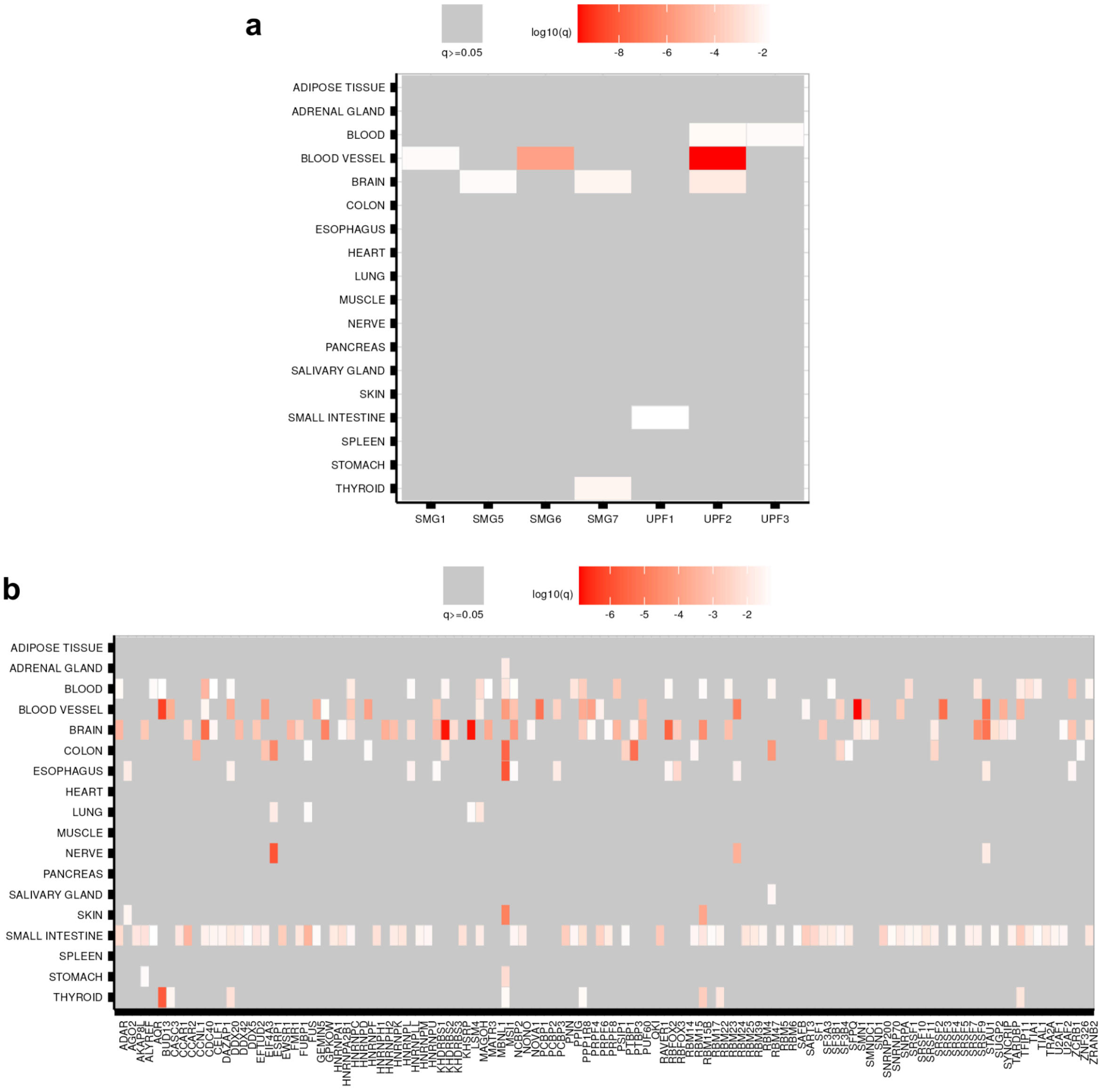

Supplementary Fig. 18. **Q-values produced by the linear regression models built to test the age effect in the expression levels of 5 essential NMD genes (a) and 107 RBPs (b) across the samples of each GTEx tissue.** Gene expression levels were measured using each gene's TPM level in each of the samples of each GTEx tissue evaluated. The independent variable to predict per linear model corresponded to each gene's TPM value in log10 scale. The dependent variables corresponded to a set of covariates providing information about the sample: age, center, gebtch, gebtchd, nabtc, nabtchd, nabtcht, hhrdy, sex and rin. P-values produced by each linear model were corrected for multiple testing using the Benjamini-Hochberg method, producing q values. Q-values have been converted to the log10 scale to facilitate the visual interpretation of the data. Grey values indicate either i) no significant effect between the sample covariate "age" and the TPM value of the gene analysed or ii) a positive coefficient assigned to the sample covariate "age" in the prediction of the TPM value of the gene analysed (indicating that higher age values are associated with higher expression levels for that gene in that sample).

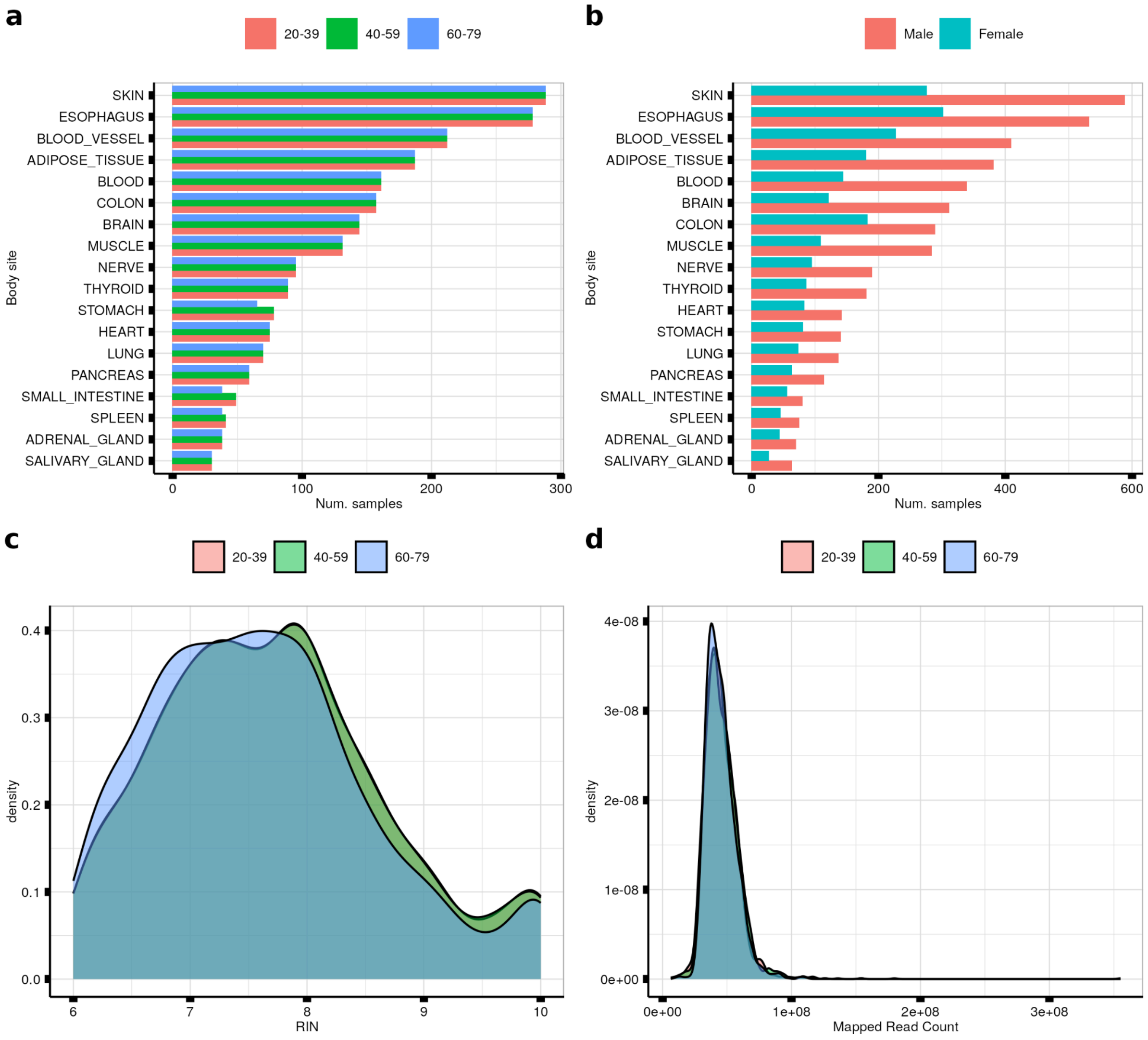

Supplementary Fig. 19. Metadata of the samples included in the "Age Stratification" intron database. All samples considered were selected after subsampling and balancing them to meet RIN number similarity across the three age groups.

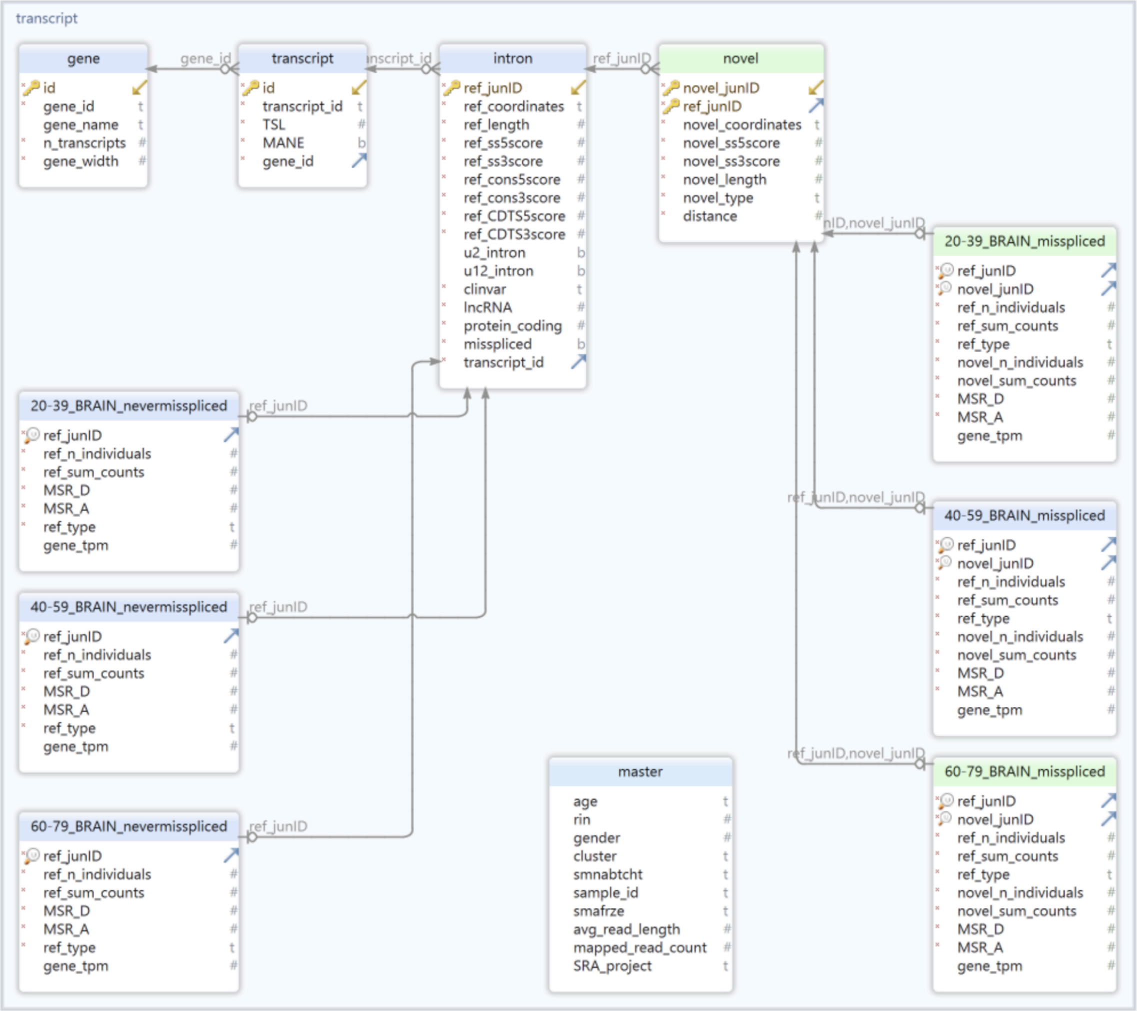

Supplementary Fig. 20. SQL schema of the "Age Stratification" intron database. To facilitate the visualisation of the database structure, only tables from the brain tissue are shown. Image generated using the software DbSchema 9.2.1 build 230214 (https://dbschema.com/).

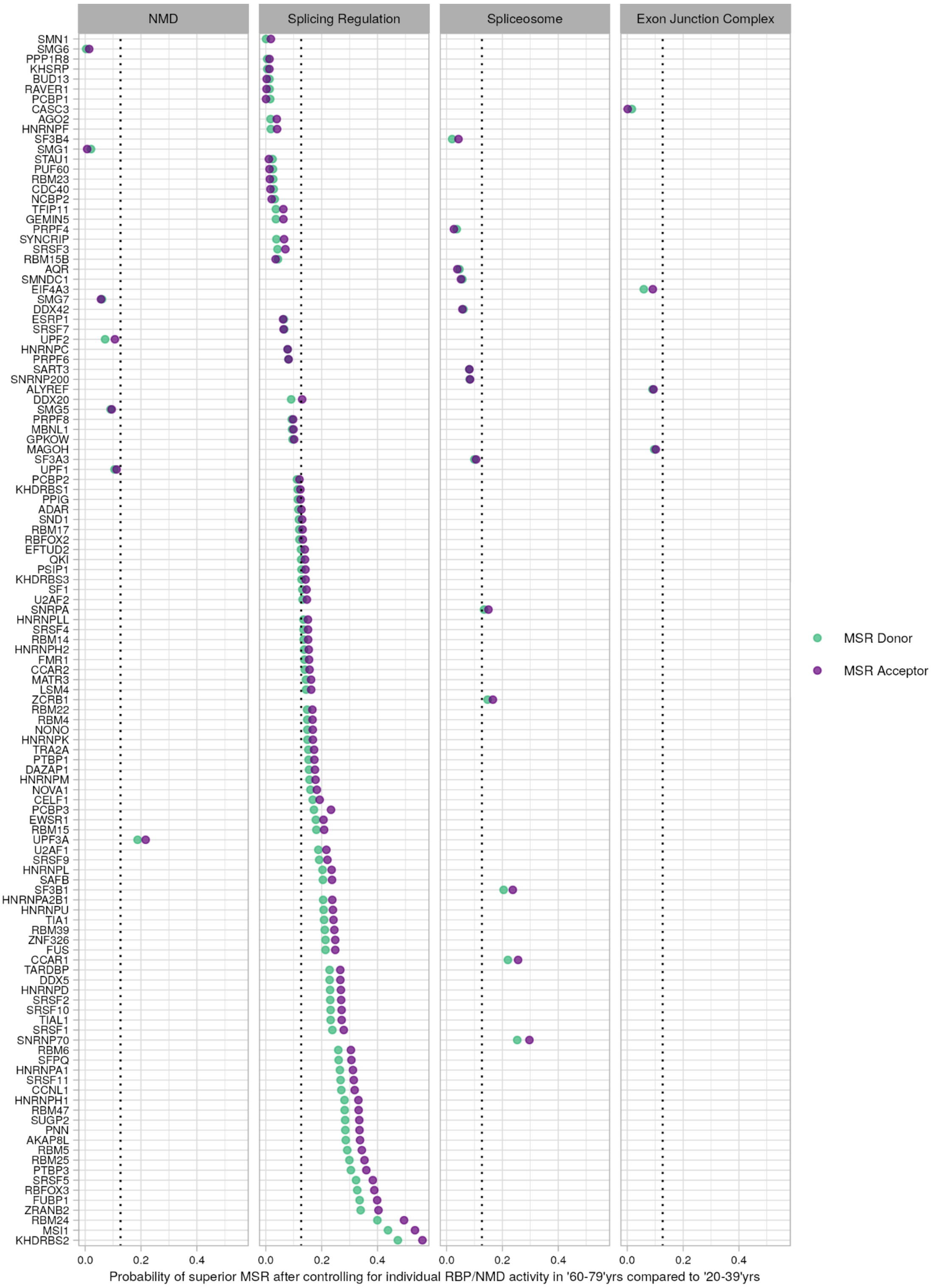

Supplementary Fig. 21. Probability of superior normalised MSRs at the 5'ss and 3'ss of the annotated introns in samples from individuals aged between 60-79 years old as compared to 20-39 yrs in blood vessel tissue. MSR values were first normalised after accounting for individual differences in fold-change TPM values of each RBP/NMD gene studied between the two age groups. The dashed vertical line represents the level of age-related splicing effects found in blood vessel tissue before RBP/NMD factor correction (Fig7.a). One-tailed Wilcoxon effect size for one-sample test (Wilcoxon one-sample signed-rank test) was performed to compare differences in normalised MSR values between the two age groups. P-values produced by each linear model were corrected for multiple testing using the Benjamini-Hochberg method, producing q values. RBP functional categorisation was extracted from (Van Nostrand et al. 2020).

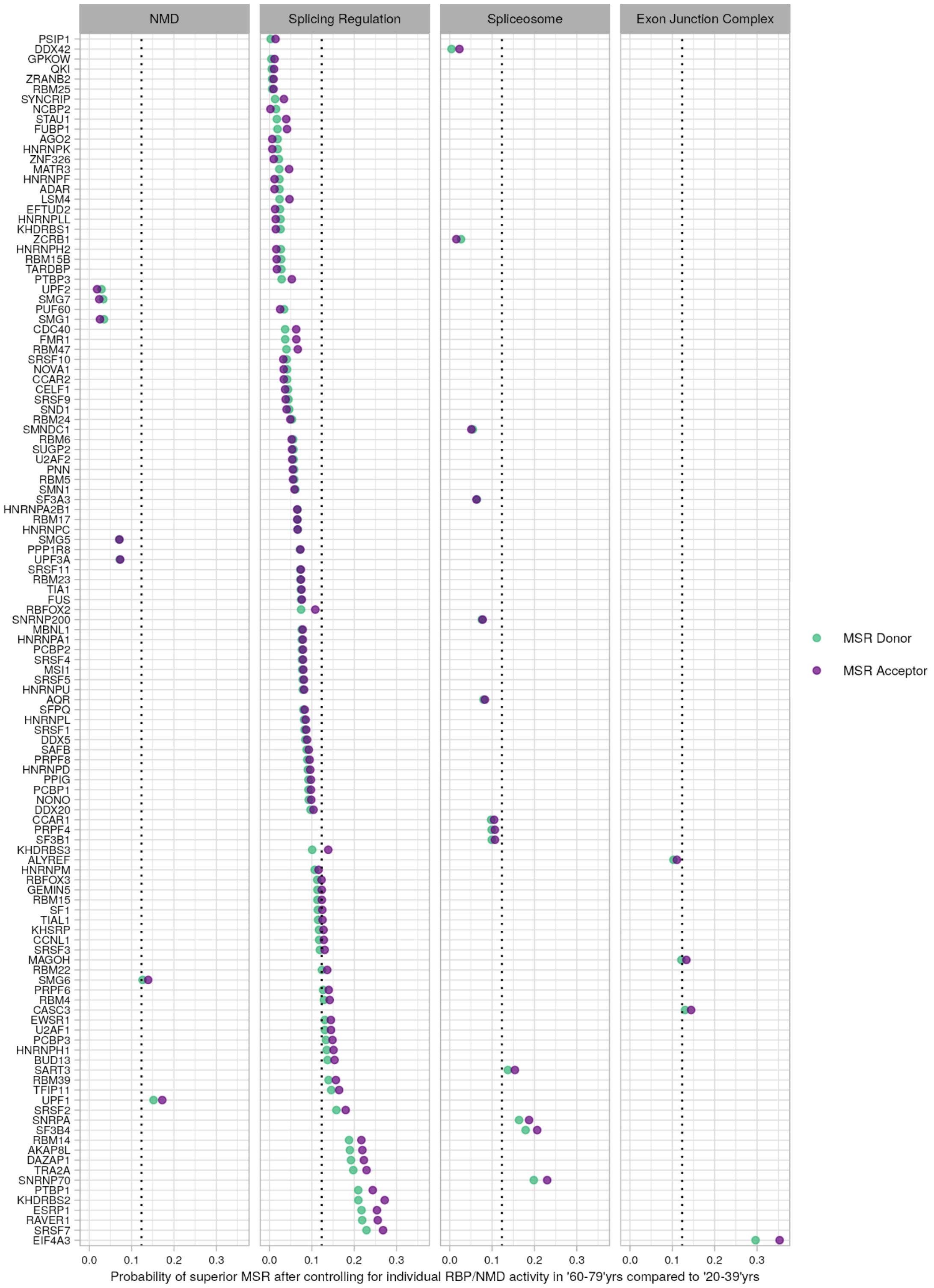

Supplementary Fig. 22. Probability of superior normalised MSRs at the 5'ss and 3'ss of the annotated introns in samples from individuals aged between 60-79 years old as compared to 20-39 yrs in brain tissue. MSR values were normalised after accounting for individual differences in fold-change TPM values between the two age groups and corresponding to the expression levels of each RBP/NMD gene studied. The dashed vertical line represents the level of age-related splicing effects found in brain tissue before RBP/NMD factor correction (Fig7.a). One-tailed Wilcoxon effect size for one-sample test (Wilcoxon one-sample signed-rank test) was performed to compare differences in normalised MSR values between the two age groups. P-values produced by each linear model were corrected for multiple testing using the Benjamini-Hochberg method, producing q values. RBP functional categorisation was extracted from (Van Nostrand et al. 2020).

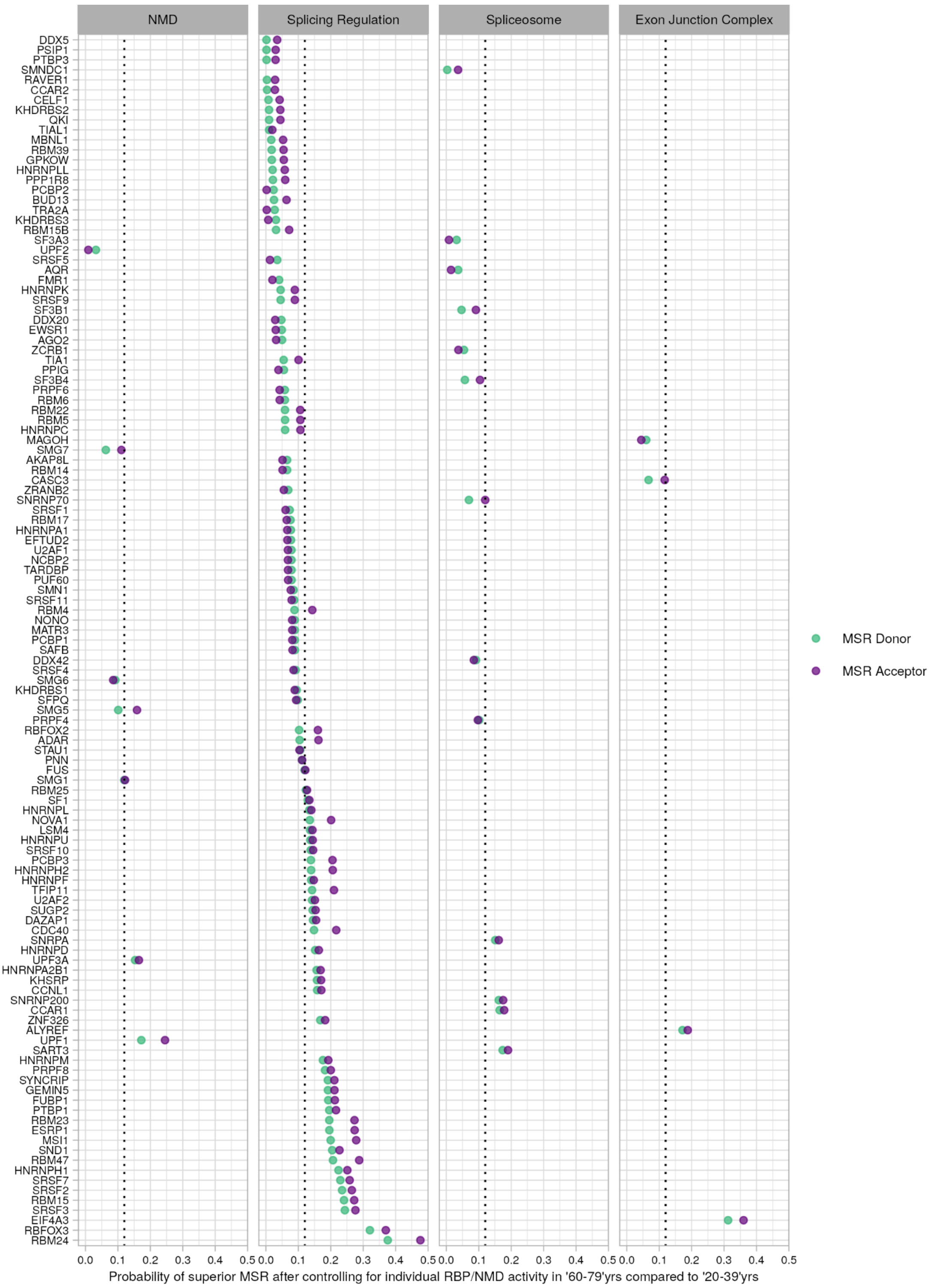

Supplementary Fig. 23. Probability of superior MSRs at the 5'ss and 3'ss of the annotated introns in samples from individuals aged between 60-79 years old as compared to 20-39 yrs in blood tissue. MSR values were normalised after accounting for individual differences in fold-change TPM values between the two age groups and corresponding to the expression levels of each RBP/NMD gene studied. The dashed vertical line represents the level of age-related splicing effects found in blood tissue before RBP/NMD factor correction (Fig7.a). One-tailed Wilcoxon effect size for one-sample test (Wilcoxon one-sample signed-rank test) was performed to compare differences in normalised MSR values between the two age groups. P-values produced by each linear model were corrected for multiple testing using the Benjamini-Hochberg method, producing q values. RBP functional categorisation was extracted from (Van Nostrand et al. 2020)

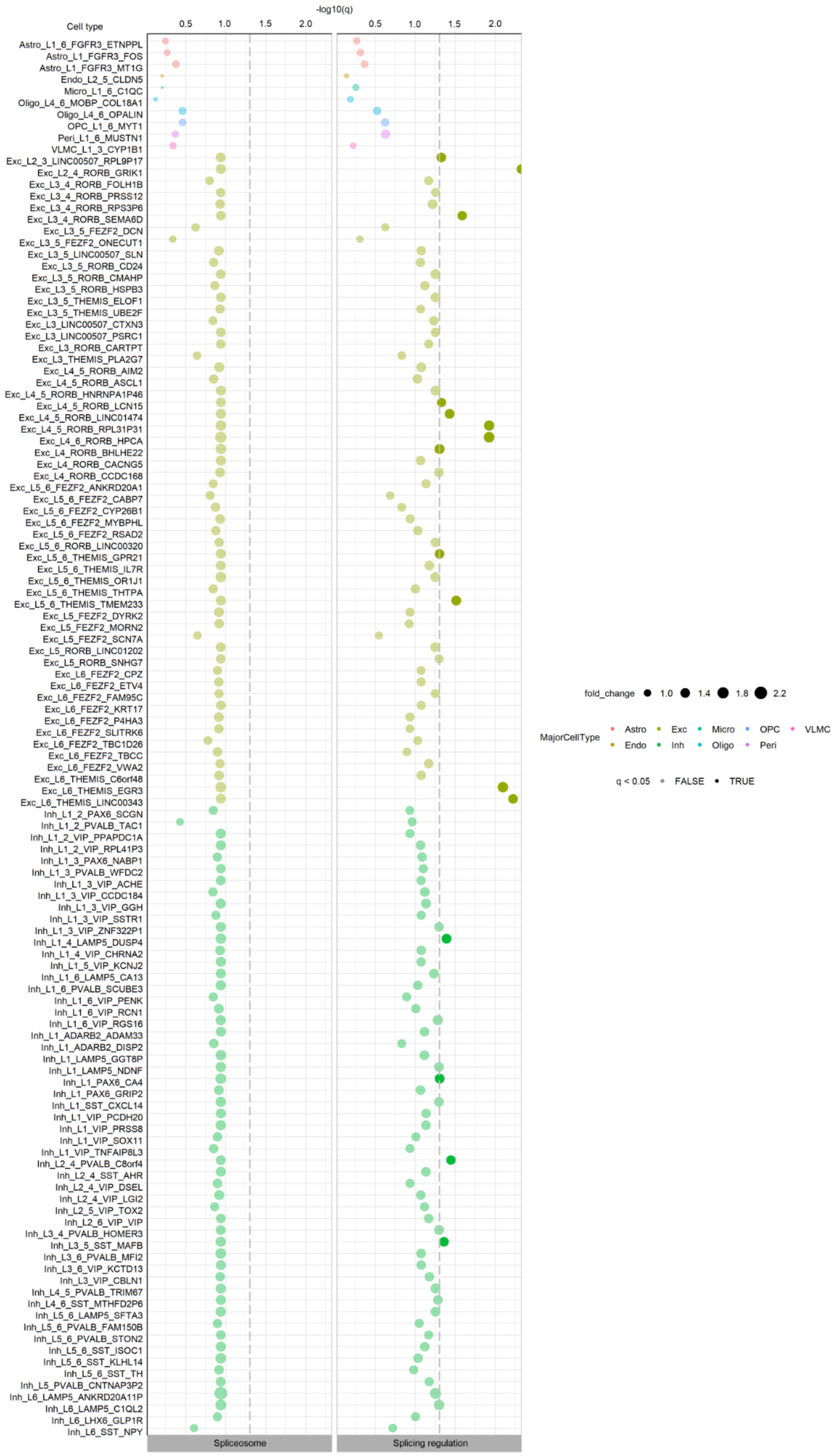

Supplementary Fig. 24. Cell-type specific expression of 98 splicing-regulators and 35 spliceosomal RBPs, defined by Van Nostrand et al. 2020, in multiple cortical regions of the human brain. The cell type annotations used correspond to the original clusters defined by the Allen Brain Atlas (Shen et al. 2012). The dashed grey vertical lines represent the minimum level of significance, with dots displayed on the right of the dashed line showing a significant expression for a given cell type. P-values were corrected for multiple testing using the Benjamini-Hochberg method, resulting in q-values.

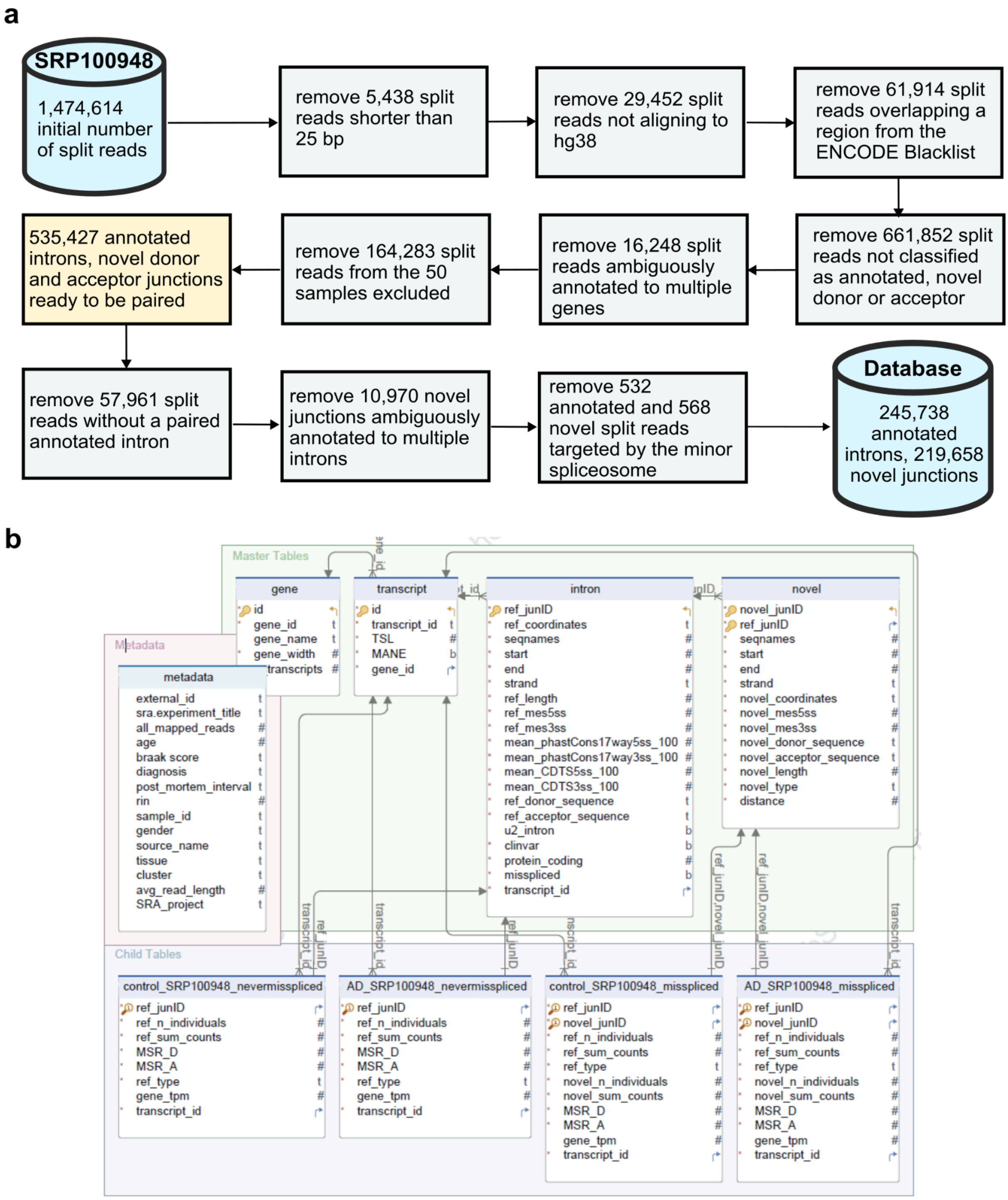

Supplementary Fig. 25. Generation of the AD/control splicing database. a. Overview of the quality-control steps applied to the dataset of split reads provided by the recount3 project ID SRP100948. **b.** SQL schema of the database built. SQL schema was generated using the software DbSchema 9.2.1 build 230214 (https://dbschema.com/).

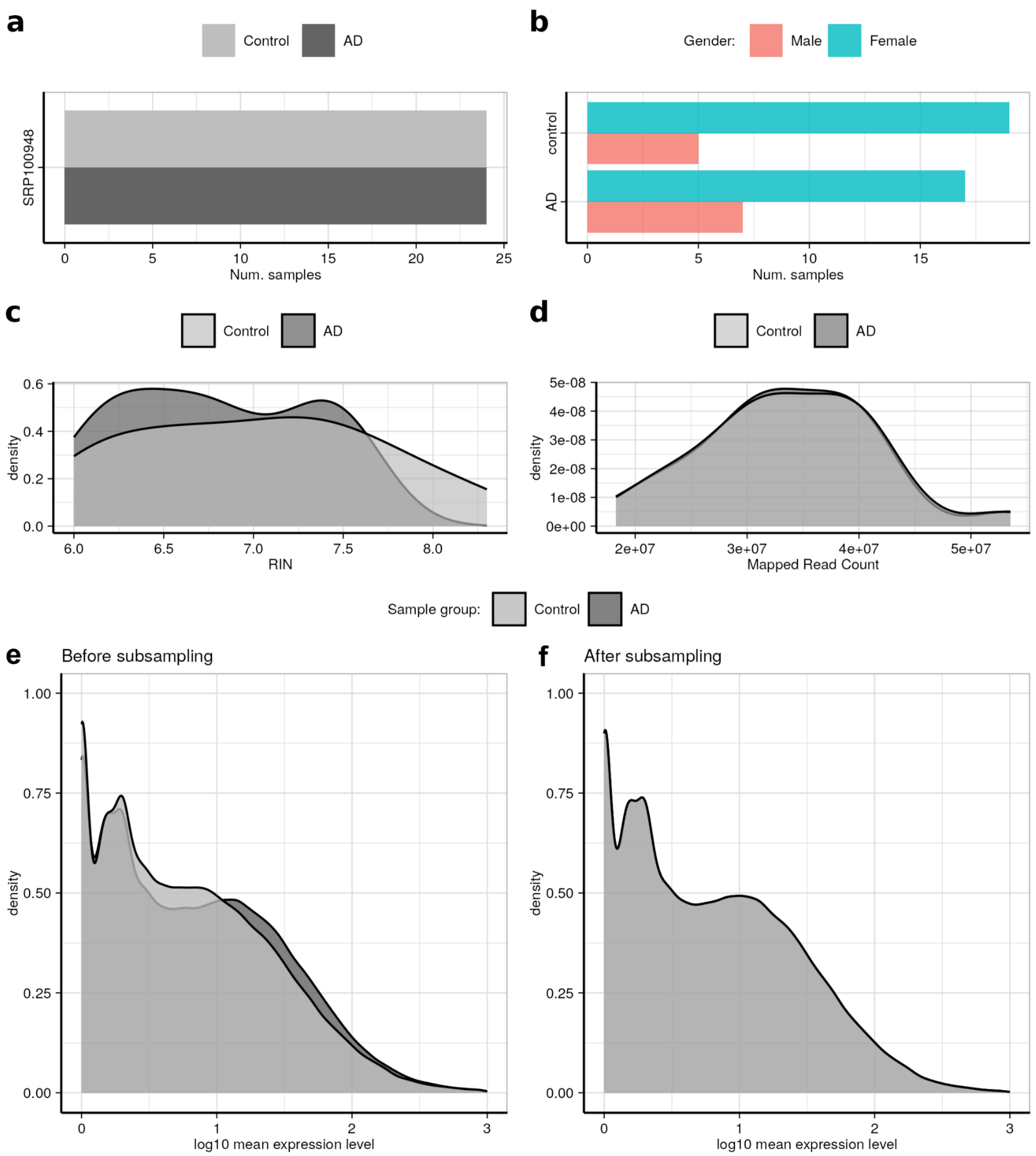

Supplementary Fig. 26. Overview of the sample metadata and intron expression levels corresponding to samples diagnosed with Alzheimer's and unaffected individuals. a. Number of samples included in the AD and control group. **b.** Distribution of samples by diagnostic (i.e. AD and control) and gender. **c.** Distribution of RIN number of the samples included in each AD and control cluster. **d.** Distribution of mapped read depth of the samples included in each AD and control cluster. **e,f.** Mean expression level of the annotated introns studied **(e)** before and **(f)** after subsampling them to match by mean expression similarity (MatchIt R package, *matchit()* function). Only samples matching read depth similarity between the AD and control clusters were included in this analysis.

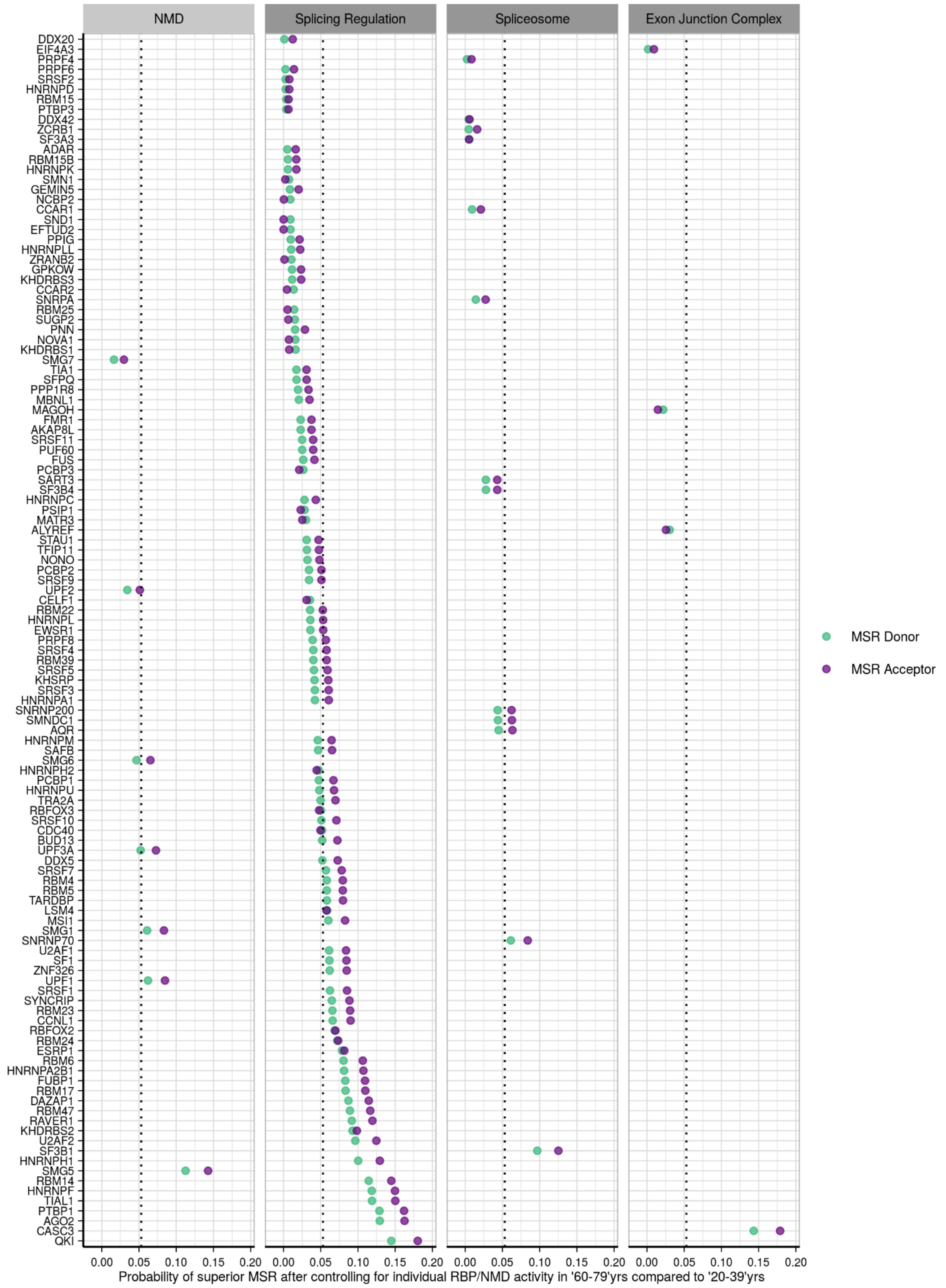

Supplementary Fig. 27. Probability of superior MSRs at the 5'ss and 3'ss of the annotated introns in samples from individuals affected with AD as compared to unaffected individuals. MSR values were normalised after accounting for individual differences in fold-change TPM values between 'AD' and 'control' groups and corresponding to the expression level of each RBP/NMD gene studied. The dashed vertical line represents the level of AD-related splicing effects found in AD/control dataset before RBP/NMD factor correction**.** One-tailed Wilcoxon effect size for one-sample test (Wilcoxon one-sample signed-rank test) was performed to compare differences in normalised MSR values between the two sample groups. P-values produced by each linear model were corrected for multiple testing using the Benjamini-Hochberg method, producing q values. RBP functional categorisation was extracted from (Van Nostrand et al. 2020). AD/control dataset corresponded to GEO:GSE95587.

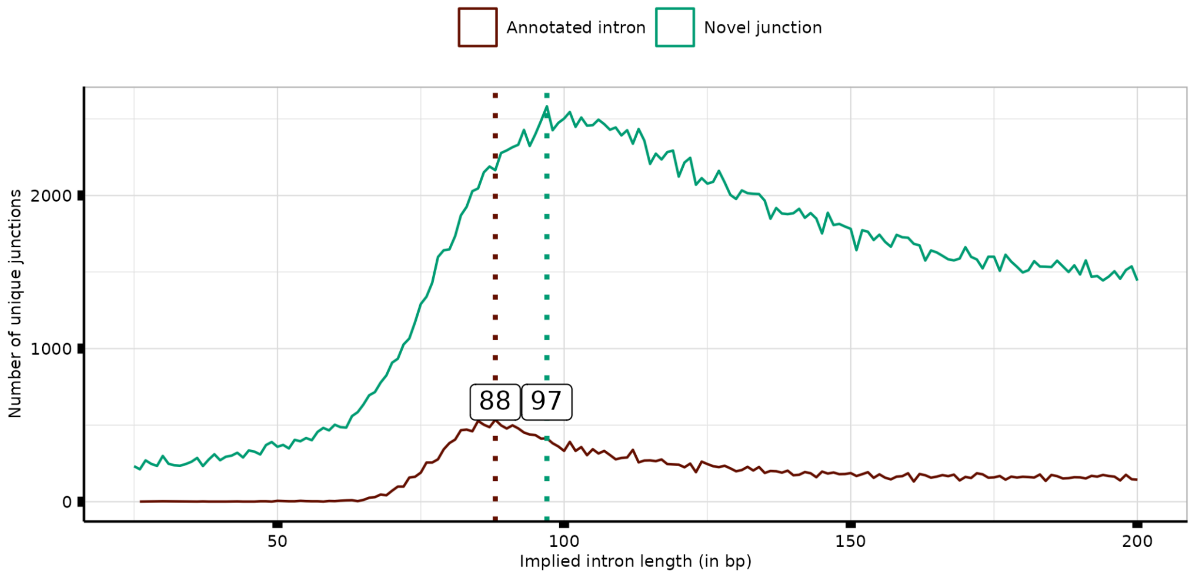

Supplementary Fig. 28. Implied intron length of the annotated and novel split reads studied. Annotated introns presented a mode length of 88 bp. Novel donor and novel acceptor junctions were collectively considered as novel junctions and presented a mode length of 97 bp.

Supplementary Table 1. Summary of median number of read counts per junction category across the samples of each GTEx tissue.

GTEx Tissue	Annotated junctions median read count	Novel Donor junctions median read count	Novel Acceptor median read count
Adipose - Subcutaneous	14575	3	3
Adipose - Visceral (Omentum)	10880	3	2
Adrenal Gland	4567	2	2
Whole Blood	10558	3	3
Cells - EBV-transformed lymphocytes	7457	2	2
Artery - Tibial	15003	3	3
Artery - Aorta	8669	3	2
Artery - Coronary	4549	2	2
Brain - Hippocampus	1842	2	2
Brain - Cortex	3177	2	2
Brain - Putamen (basal ganglia)	1972	2	2
Brain - Anterior cingulate cortex (BA24)	1849	2	2
Brain - Cerebellar Hemisphere	4675	2	2
Brain - Frontal Cortex (BA9)	2695	2	2
Brain - Spinal cord (cervical c-1)	1753	2	2
Brain - Substantia nigra	1151	2	2
Brain - Nucleus accumbens (basal ganglia)	3106	2	2
Brain - Hypothalamus	2243	2	2
Brain - Cerebellum	4519	2	2
Brain - Caudate (basal ganglia)	2893	2	2
Brain - Amygdala	1337	2	2
Colon - Transverse	7523	2	2
Colon - Sigmoid	6418	2	2
Esophagus - Muscularis	9817	3	2
Esophagus - Mucosa	10702	2	2
Esophagus - Gastroesophageal Junction	6791	2	2
Heart - Atrial Appendage	6635	2	2
Heart - Left Ventricle	5170	2	2
Lung	12046	3	2
Muscle - Skeletal	15316	3	2
Nerve - Tibial	12931	3	3
Pancreas	3346	2	2
Minor Salivary Gland	3165	2	2
Cells - Cultured fibroblasts	16767	2	2
Skin - Sun Exposed (Lower leg)	14728	3	3
Skin - Not Sun Exposed (Suprapubic)	11682	3	3
Small Intestine - Terminal Ileum	3596	2	2
Spleen	3609	2	2
Stomach	5225	2	2
Thyroid	12729	3	3

Supplementary Table 2. Summary of mode distances (in bp) from each novel donor and acceptor junction to their paired annotated intron.

GTEx Tissue	Novel donor junctions downstream reference 5’ss	Novel donor junctions upstream reference 5’ss	Novel acceptor junctions upstream ref 3’ss	Novel acceptor junctions downstream ref 3’ss
Adipose - Subcutaneous	-4	3	-22	2
Adipose - Visceral (Omentum)	-4	3	-21	2
Adrenal Gland	-4	3	-21	2
Whole Blood	-4	3	-21	2
Cells - EBV-transformed lymphocytes	-4	3	-21	2
Artery - Tibial	-4	3	-24	2
Artery - Aorta	-4	3	-24	2
Artery - Coronary	-4	3	-21	4
Brain - Hippocampus	-4	3	-21	4
Brain - Cortex	-4	3	-21	4
Brain - Putamen (basal ganglia)	-4	3	-21	4
Brain - Anterior cingulate cortex (BA24)	-4	3	-21	4
Brain - Cerebellar Hemisphere	-4	3	-21	4
Brain - Frontal Cortex (BA9)	-4	3	-21	4
Brain - Spinal cord (cervical c-1)	-4	4	-21	4
Brain - Substantia nigra	-4	4	-21	4
Brain - Nucleus accumbens(basal ganglia)	-4	3	-21	4
Brain - Hypothalamus	-4	3	-21	4
Brain - Cerebellum	-4	3	-22	4
Brain - Caudate (basal ganglia)	-4	3	-21	4
Brain - Amygdala	-4	4	-21	4
Colon - Transverse	-4	3	-21	2
Colon - Sigmoid	-4	3	-21	2
Esophagus - Muscularis	-4	3	-21	2
Esophagus - Mucosa	-4	3	-21	2
Esophagus - Gastroesophageal Junction	-4	3	-21	2
Heart - Atrial Appendage	-4	3	-21	2
Heart - Left Ventricle	-4	3	-21	2
Lung	-4	3	-21	2
Muscle - Skeletal	-4	3	-21	2
Nerve - Tibial	-4	3	-22	2
Pancreas	-4	3	-21	4
Minor Salivary Gland	-4	4	-21	4
Cells - Cultured fibroblasts	-4	3	-21	2
Skin - Sun Exposed (Lower leg)	-4	3	-22	2
Skin - Not Sun Exposed (Suprapubic)	-4	3	-21	2
Small Intestine - Terminal Ileum	-4	3	-21	4
Spleen	-4	3	-21	4
Stomach	-4	3	-21	2
Thyroid	-4	3	-21	2

Mode distances (in base pairs) calculated from each novel splice junction to its paired annotated intron across the samples from each GTEx tissue.

Supplementary Table 3. Summary of the one-sided paired Wilcoxon signed rank test output run to test three alternative hypotheses (T.1, T.2 and T.3) regarding the level of mis-splicing activity detected across the samples of each GTEx tissue.

GTEx Tissue	Statistical Test (H1)	Effect Size	p-value
Adipose - Subcutaneous	T.1	0.074	1.19E-35
	T.2	0.154	1.89E-151
	T.3	0.168	5.96E-198
Adipose - Visceral (Omentum)	T.1	0.078	1.30E-40
	T.2	0.147	6.54E-144
	T.3	0.155	2.94E-173
Adrenal Gland	T.1	0.055	1.08E-24
	T.2	0.17	9.90E-164
	T.3	0.197	9.08E-233
Whole Blood	T.1	0.085	1.91E-43
	T.2	0.219	0
	T.3	0.235	0
Cells - EBV-transformed lymphocytes	T.1	0.059	4.19E-25
	T.2	0.253	0
	T.3	0.283	0
Artery - Tibial	T.1	0.078	5.40E-43
	T.2	0.163	2.78E-165
	T.3	0.184	9.61E-228
Artery - Aorta	T.1	0.083	2.67E-42
	T.2	0.15	5.89E-150
	T.3	0.159	1.17E-190
Artery - Coronary	T.1	0.054	9.92E-33
	T.2	0.141	1.14E-113
	T.3	0.165	4.60E-178
Brain - Hippocampus	T.1	0.059	1.11E-27
	T.2	0.157	5.01E-130
	T.3	0.187	3.71E-190
Brain - Cortex	T.1	0.061	2.05E-25
	T.2	0.158	1.97E-146
	T.3	0.176	1.21E-195
Brain - Putamen (basal ganglia)	T.1	0.059	4.28E-26
	T.2	0.177	5.09E-183
	T.3	0.206	1.98E-252
Brain - Anterior cingulate cortex (BA24)	T.1	0.056	2.23E-19
	T.2	0.18	3.39E-171
	T.3	0.196	1.04E-218
Brain - Cerebellar Hemisphere	T.1	0.089	4.03E-43
	T.2	0.202	8.65E-237
	T.3	0.225	0
Brain - Frontal Cortex (BA9)	T.1	0.064	1.50E-25
	T.2	0.171	4.02E-173
	T.3	0.194	2.54E-241
Brain - Spinal cord (cervical c-1)	T.1	0.054	1.08E-21
	T.2	0.168	1.53E-140
	T.3	0.198	3.27E-204
Brain - Substantia nigra	T.1	0.06	1.51E-23
	T.2	0.2	3.64E-190
	T.3	0.216	1.64E-229

Brain - Nucleus accumbens(basal ganglia)	T.1	0.062	2.34E-24
	T.2	0.172	6.67E-173
	T.3	0.19	1.69E-228
Brain - Hypothalamus	T.1	0.054	1.46E-20
	T.2	0.165	4.73E-152
	T.3	0.19	7.61E-212
Brain - Cerebellum	T.1	0.059	4.42E-27
	T.2	0.172	2.84E-180
	T.3	0.21	2.78E-289
Brain - Caudate (basal ganglia)	T.1	0.063	2.04E-23
	T.2	0.175	6.77E-175
	T.3	0.194	1.86E-240
Brain - Amygdala	T.1	0.054	3.33E-19
	T.2	0.158	1.04E-137
	T.3	0.182	3.87E-180
Colon - Transverse	T.1	0.063	3.78E-31
	T.2	0.142	5.91E-121
	T.3	0.166	2.72E-181
Colon - Sigmoid	T.1	0.072	2.93E-37
	T.2	0.144	2.87E-126
	T.3	0.151	5.85E-157
Esophagus - Muscularis	T.1	0.074	6.43E-44
	T.2	0.139	6.04E-118
	T.3	0.152	1.56E-165
Esophagus - Mucosa	T.1	0.071	4.46E-25
	T.2	0.121	8.58E-100
	T.3	0.138	2.24E-133
Esophagus - Gastroesophageal Junction	T.1	0.069	4.28E-37
	T.2	0.147	3.37E-137
	T.3	0.179	1.84E-207
Heart - Atrial Appendage	T.1	0.072	3.66E-32
	T.2	0.137	1.99E-125
	T.3	0.145	4.11E-145
Heart - Left Ventricle	T.1	0.066	1.72E-26
	T.2	0.136	2.68E-119
	T.3	0.149	7.10E-158
Lung	T.1	0.069	5.27E-38
	T.2	0.13	9.79E-114
	T.3	0.159	1.20E-178
Muscle - Skeletal	T.1	0.068	1.84E-36
	T.2	0.135	3.50E-139
	T.3	0.159	1.17E-204
Nerve - Tibial	T.1	0.071	1.77E-36
	T.2	0.155	1.43E-161
	T.3	0.169	3.00E-221
Pancreas	T.1	0.054	2.08E-22
	T.2	0.166	5.40E-173
	T.3	0.19	1.53E-240
Minor Salivary Gland	T.1	0.066	1.20E-31
	T.2	0.137	1.24E-94
	T.3	0.155	1.18E-138
Cells - Cultured fibroblasts	T.1	0.069	3.12E-31
	T.2	0.195	7.33E-241

	T.3	0.212	0
Skin - Sun Exposed (Lower leg)	T.1	0.074	9.02E-29
	T.2	0.144	1.16E-129
	T.3	0.149	2.16E-160
Skin - Not Sun Exposed (Suprapubic)	T.1	0.071	1.86E-32
	T.2	0.121	1.96E-97
	T.3	0.143	6.02E-160
Small Intestine - Terminal Ileum	T.1	0.055	1.20E-22
	T.2	0.143	4.23E-112
	T.3	0.167	6.16E-165
Spleen	T.1	0.063	6.51E-31
	T.2	0.188	2.03E-216
	T.3	0.211	1.67E-283
Stomach	T.1	0.071	1.49E-33
	T.2	0.13	5.20E-103
	T.3	0.143	3.36E-137
Thyroid	T.1	0.082	3.23E-42
	T.2	0.154	4.78E-165
	T.3	0.169	9.63E-222

T.1 = H1: Annotated introns are less frequently mis-spliced at their donor than at their acceptor splice site; T.2 = H1: Annotated introns from non-coding transcripts are more frequently mis-spliced at their donor splice site than introns from protein-coding transcripts; T.3 = H1: Annotated introns from non-coding transcripts are more frequently mis-spliced at their acceptor splice sites than introns from PC transcripts.

Supplementary Table 4. Summary of the one-sided paired Wilcoxon signed rank tests run to evaluate whether the knockdown of each RBP/NMD factor had a greater effect on inaccurate splicing levels at the 5'ss (i.e. MSR Donor) of the introns studied compared with untreated control samples.

Target RBP/NMD factor	p-value	FDR	Effect Size	Category
ADAR	1.06E-32	1.59E-32	0.036	Splicing regulation
AQR	0	0	0.388	Spliceosome
BUD13	0.575	0.597115385	0.001	Splicing regulation
CELF1	3.94E-87	8.18308E-87	0.061	Splicing regulation
DAZAP1	0.00141	0.00158625	0.006	Splicing regulation
EFTUD2	0	0	0.167	Splicing regulation
EIF4G1	3.26E-23	4.51385E-23	0.028	NMD
EWSR1	0.353	0.373764706	0.002	Splicing regulation
FUBP1	5.34E-148	1.602E-147	0.085	Splicing regulation
GEMIN5	2.23E-94	5.23565E-94	0.066	Splicing regulation
GPKOW	1.46E-139	3.7543E-139	0.073	Splicing regulation
HNRNPC	0	0	0.183	Splicing regulation
HNRNPU	7.14E-61	1.32952E-60	0.048	Splicing regulation
KHDRBS1	8.59E-53	1.49632E-52	0.045	Splicing regulation
KHSRP	1.41E-156	4.4788E-156	0.085	Splicing regulation
MAGOH	0	0	0.227	Exon Junction Complex
MATR3	2.77E-142	7.479E-142	0.082	Splicing regulation
NCBP2	8.55E-223	3.8475E-222	0.102	Splicing regulation
NONO	6.46E-52	1.09012E-51	0.054	Splicing regulation
PABPC1	1.45E-249	8.7E-249	0.112	NMD
PCBP1	2.28E-38	3.51771E-38	0.046	Splicing regulation
PCBP2	2.35E-216	9.7615E-216	0.1	Splicing regulation
PPIG	2.72E-09	3.264E-09	0.021	Splicing regulation
PRPF4	2.16E-61	4.16571E-61	0.057	Spliceosome
PRPF6	4.98E-26	7.26811E-26	0.033	Splicing regulation
PSIP1	1.57E-42	2.49353E-42	0.04	Splicing regulation
PTBP1	9.57E-121	2.349E-120	0.08	Splicing regulation
PUF60	0	0	0.113	Splicing regulation
QKI	5.39E-92	1.16424E-91	0.062	Splicing regulation
RAVER1	3.42E-227	1.8468E-226	0.097	Splicing regulation
RBM15	4.39E-12	5.51302E-12	0.019	Splicing regulation
RBM22	1.47E-253	9.9225E-253	0.117	Spliceosome
RBM39	1.69E-08	1.98391E-08	0.025	Spliceosome
RPS10	0.00491	0.00541102	0.01	NMD
RPS19	7.06E-85	1.412E-84	0.073	NMD
RPS3A	1.72E-16	2.322E-16	0.031	NMD
SAFB2	0.993	0.993	0.005	Novel RBP
SART3	2.87E-23	4.07842E-23	0.031	Spliceosome
SF1	1.75E-08	2.01064E-08	0.026	Spliceosome
SF3A3	0	0	0.195	Spliceosome
SF3B4	1.44E-224	7.0691E-224	0.106	Spliceosome
SMN1	8.91E-15	1.14557E-14	0.029	Spliceosome
SMNDC1	0.694	0.70709434	0	Spliceosome
SND1	0.129	0.13932	0.006	Splicing regulation
SNRNP200	4.19E-206	1.6161E-205	0.113	Spliceosome
SRSF1	5.53E-182	1.8664E-181	0.093	Splicing regulation
SUGP2	2.33E-53	4.194E-53	0.053	Splicing regulation

TARDBP	1.08E-146	3.0695E-146	0.075	Splicing regulation
TIAL1	9.20E-49	1.50545E-48	0.039	Splicing regulation
U2AF1	0	0	0.12	Spliceosome
U2AF2	2.36E-192	8.496E-192	0.096	Spliceosome
UPF1	2.34E-94	5.265E-94	0.063	NMD
UPF2	1.61E-15	2.12049E-15	0.026	NMD
ZRANB2	3.20E-10	3.92727E-10	0.021	Splicing regulation

H0 = The MSR_Donor values of the common introns between knockdown and control samples are symmetric about their median value. H1 = The MSR_Donor values of the common introns between knockdown and control samples are greater in knockdown samples at their median value than in control samples. p-values were adjusted using the False Discovery Rate (FDR) method.

Supplementary Table 5. Summary of the one-sided paired Wilcoxon signed rank tests run to evaluate whether the knockdown of each RBP/NMD factor had a greater effect on inaccurate splicing levels at the 3'ss (i.e. MSR Acceptor) of the introns studied compared with untreated control samples.

Target RBP/NMD factor	p-value	FDR	Effect Size	Category
ADAR	5.45E-80	9.53E-80	0.063	Splicing regulation
AQR	0	0	0.639	Spliceosome
BUD13	8.07E-04	8.71E-04	0.017	Splicing regulation
CELF1	4.64E-107	1.01E-106	0.074	Splicing regulation
DAZAP1	0.0368	0.037101639	0.004	Splicing regulation
EFTUD2	0	0	0.142	Splicing regulation
EIF4G1	3.86E-18	4.72E-18	0.024	NMD
EWSR1	5.92E-06	6.54E-06	0.013	Splicing regulation
FUBP1	2.13E-277	8.19E-277	0.118	Splicing regulation
GEMIN5	1.53E-188	4.12E-188	0.096	Splicing regulation
GPKOW	5.93E-134	1.42E-133	0.08	Splicing regulation
HNRNPC	0	0	0.204	Splicing regulation
HNRNPU	3.52E-60	5.35E-60	0.047	Splicing regulation
KHDRBS1	1.13E-62	1.84E-62	0.054	Splicing regulation
KHSRP	1.37E-197	4.18E-197	0.092	Splicing regulation
MAGOH	0	0	0.261	Exon Junction Complex
MATR3	2.62E-193	7.49E-193	0.091	Splicing regulation
NCBP2	1.24E-271	4.09E-271	0.108	Splicing regulation
NONO	5.15E-99	1.07E-98	0.075	Splicing regulation
PABPC1	0	0	0.139	NMD
PCBP1	3.20E-81	5.82E-81	0.061	Splicing regulation
PCBP2	3.43E-84	6.49E-84	0.057	Splicing regulation
PPIG	4.27E-33	5.53E-33	0.043	Splicing regulation
PRPF4	6.55E-58	9.63E-58	0.068	Spliceosome
PRPF6	9.78E-04	0.001031091	0.007	Splicing regulation
PSIP1	4.90E-41	6.72E-41	0.04	Splicing regulation
PTBP1	7.79E-157	1.97E-156	0.082	Splicing regulation
PUF60	0	0	0.152	Splicing regulation
QKI	1.30E-119	2.96E-119	0.07	Splicing regulation
RAVER1	1.42E-267	4.64E-267	0.105	Splicing regulation
RBM15	4.19E-53	6.14E-53	0.044	Splicing regulation
RBM22	0	0	0.164	Spliceosome
RBM39	1.05E-50	1.48E-50	0.053	Spliceosome
RPS10	0.0052	0.005359777	0.009	NMD
RPS19	1.10E-62	1.84E-62	0.063	NMD
RPS3A	8.28E-07	9.37E-07	0.021	NMD
SAFB2	0.993	0.993	0.006	Novel RBP
SART3	4.32E-16	5.14E-16	0.026	Spliceosome
SF1	7.33E-61	1.15E-60	0.058	Spliceosome
SF3A3	0	0	0.303	Spliceosome
SF3B4	0	0	0.216	Spliceosome
SMN1	1.15E-25	1.44E-25	0.032	Spliceosome
SMNDC1	5.26E-08	6.10E-08	0.015	Spliceosome
SND1	0.56	0.561521739	0.004	Splicing regulation
SNRNP200	1.82E-274	6.46E-274	0.123	Spliceosome
SRSF1	3.77E-165	1.01E-164	0.085	Splicing regulation
SUGP2	1.41E-51	2.06E-51	0.051	Splicing regulation

TARDBP	1.20E-92	2.47E-92	0.057	Splicing regulation
TIAL1	8.98E-33	1.16E-32	0.03	Splicing regulation
U2AF1	0	0	0.2	Spliceosome
U2AF2	0	0	0.192	Spliceosome
UPF1	5.04E-88	9.95E-88	0.068	NMD
UPF2	2.92E-39	3.89E-39	0.04	NMD
ZRANB2	0.549	0.551991826	0.001	Splicing regulation

H0 = The MSR_Acceptor values of the common introns between knockdown and control samples are symmetric about their median value. H1 = The MSR_Acceptor values of the common introns between knockdown and control samples are greater in knockdown samples at their median value than in control samples. p-values were adjusted using the False Discovery Rate (FDR) method.

Supplementary Table 6. Summary of the Pearson's Chi-squared test to evaluate whether introns with the increasing levels of MSR values at each splice site under knockdown conditions of a given RBP also presented higher densities of binding sites for that RBP.

RBP	MSR Type	Introns increasing MSR / w CLIP sites	Introns not increasing MSR / w CLIP sites	Introns increasing MSR / no CLIP sites	Introns not increasing MSR / no CLIP sites	chi-sq statistic	chi-sq p-value
HNRNPC	MSR_D	4862	13883	7821	83213	4575.483803	0
HNRNPC	MSR_A	5311	20036	7372	77060	2848.548043	0
HNRNPU	MSR_D	1968	12988	5124	89699	1284.331085	2.87E-281
HNRNPU	MSR_A	2253	17837	4839	84850	918.8181218	7.96E-202
KHDRBS1	MSR_D	750	12032	2426	94571	454.4073543	7.92E-101
KHDRBS1	MSR_A	946	17010	2230	89593	430.1321882	1.52E-95
NONO	MSR_D	1267	13624	2912	91976	1038.565187	7.44E-228
NONO	MSR_A	1426	19406	2753	86194	647.2216354	8.98E-143
PCBP2	MSR_D	4470	11848	14762	78699	1292.50629	4.81E-283
PCBP2	MSR_A	4756	14616	14476	75931	804.4120621	5.93E-177
PTBP1	MSR_D	5147	10708	18555	75369	1293.200463	3.40E-283
PTBP1	MSR_A	6348	14597	17354	71480	1161.215548	1.64E-254
RBM22	MSR_D	5769	13589	17099	73322	1146.166351	3.06E-251
RBM22	MSR_A	8339	19876	14529	67035	1751.931281	0
SF3A3	MSR_D	9158	13817	25152	61652	1001.313352	9.31E-220
SF3A3	MSR_A	14439	22701	19871	52768	1517.743246	0
SF3B4	MSR_D	6924	9199	32366	61290	420.6103567	1.80E-93
SF3B4	MSR_A	11740	15652	27550	54837	793.283717	1.56E-174
SMNDC1	MSR_D	4657	8068	25903	71151	549.2735072	1.81E-121
SMNDC1	MSR_A	6335	11518	24225	67701	620.0882526	7.15E-137
SND1	MSR_D	4188	8010	26973	70608	238.5244306	8.25E-54
SND1	MSR_A	5662	10602	25499	68016	387.6945411	2.63E-86
SRSF1	MSR_D	10739	5895	49370	43775	760.4620925	2.13E-167
SRSF1	MSR_A	13595	7885	46514	41785	785.1710579	9.04E-173
TARDBP	MSR_D	5838	9578	21597	72766	1586.029341	0
TARDBP	MSR_A	6934	12456	20501	69888	1456.373879	1.18E-318
U2AF1	MSR_D	9203	7656	38371	54549	1026.379468	3.31E-225
U2AF1	MSR_A	13984	12390	33590	49815	1325.696149	2.95E-290
U2AF2	MSR_D	8851	5477	44332	51119	1171.516089	9.46E-257
U2AF2	MSR_A	13917	9601	39266	46995	1379.265433	6.73E-302

From the RBPs studied in the manuscript, only 15 RBPs presented RNA/RBP binding sites data provided by the ENCORI database (Li JH, et al. Nucleic Acids Res). Inaccurate splicing at the 5'ss was evaluated through the MSR_D measure. Inaccurate splicing at the 3'ss was evaluated through the MSR_A measure. The table columns: *"Introns increasing MSR / w CLIP sites"*, *"Introns not increasing MSR / w CLIP sites"*, *"Introns increasing MSR / no CLIP sites"*, and *"Introns not increasing MSR / no CLIP sites"* contained information about the number of introns per category and were used to create a contingency table to feed the two-sided Chi-square test performed per RBP.

Supplementary Table 7. One-tailed paired Wilcoxon Rank test to evaluate whether the annotated introns evaluated per GTEx tissue present a higher frequency of inaccurate splicing activity with increasing age at each splice.

GTEx tissue	MSR Type	Effect Size	FDR	p-value
ADRENAL GLAND	MSR Donor	0.044	1.51225E-46	6.72E-47
BLOOD	MSR Donor	0.119	0	0
BLOOD VESSEL	MSR Donor	0.12	0	0
BRAIN	MSR Donor	0.117	7.6867E-305	1.7E-305
COLON	MSR Donor	0.007	0.010426437	0.006951
ESOPHAGUS	MSR Donor	0.11	0	0
HEART	MSR Donor	0.035	1.31695E-31	6.58E-32
MUSCLE	MSR Donor	0.054	1.30986E-97	5.09E-98
NERVE	MSR Donor	0.064	1.9237E-143	6.4E-144
PANCREAS	MSR Donor	0.034	8.59615E-22	5.25E-22
SALIVARY GLAND	MSR Donor	0.084	2.9554E-213	8.2E-214
SKIN	MSR Donor	0	0.516080426	0.372725
THYROID	MSR Donor	0.033	2.87509E-30	1.6E-30
ADRENAL GLAND	MSR Acceptor	0.047	1.22239E-54	6.79E-55
BLOOD	MSR Acceptor	0.121	0	0
BLOOD VESSEL	MSR Acceptor	0.133	0	0
BRAIN	MSR Acceptor	0.129	0	0
COLON	MSR Acceptor	0.04	1.09982E-46	6.72E-47
ESOPHAGUS	MSR Acceptor	0.133	0	0
HEART	MSR Acceptor	0.044	1.02089E-54	5.1E-55
MUSCLE	MSR Acceptor	0.042	3.98728E-61	1.77E-61
NERVE	MSR Acceptor	0.076	5.3866E-186	1.5E-186
PANCREAS	MSR Acceptor	0.028	1.66871E-12	1.21E-12
SALIVARY GLAND	MSR Acceptor	0.078	1.4173E-182	4.7E-183
SKIN	MSR Acceptor	0.028	2.08111E-25	1.39E-25
THYROID	MSR Acceptor	0.054	3.39577E-80	1.32E-80

Only annotated introns overlapping all age groups and tissues were used, corresponding to N=139,419. Inaccurate splicing at the 5’ss was evaluated through the “MSR Donor” measure. Inaccurate splicing at the 3’ss was evaluated through the “MSR Acceptor” measure. H0 = The introns in the '20-39' & '60-79' sample groups present distributions of “MSR_D” values that are symmetric about their median value. H1 = The introns in the '60-79' sample group present a distribution of higher “MSR_D” values compared with the 20-39 yrs group, indicating a higher frequency of inaccurate splicing activity. p-values were adjusted using the False Discovery Rate (FDR) method.

Supplementary Table 8. Ranked top 10 outcomes (ordered by ascending effect size result) corresponding from the one-tailed, paired Wilcoxon effect size test to evaluate the probability of superior normalised MSR values with increasing age across introns from blood tissue samples.

RBP/NMD factor	Effect Size	FDR (corrected p values)	MSR Type	Splicing Regulation	Spliceosome	Exon Junction Complex	NMD
SMN1	0.000525	0.15631	MSR Donor	1	1	0	0
PCBP1	0.000543	0.356563	MSR Acceptor	1	0	0	0
CASC3	0.001419	0.243219	MSR Acceptor	0	0	1	0
RAVER1	0.002333	0.831146	MSR Acceptor	1	0	0	0
BUD13	0.002944	0.902203	MSR Acceptor	1	1	0	0
SMG6	0.003588	0.018341	MSR Donor	0	0	0	1
PPP1R8	0.004795	0.005344	MSR Donor	1	0	0	0
KHSRP	0.005102	0.003792	MSR Donor	1	0	0	0
SMG1	0.00713	0.001841	MSR Acceptor	0	0	0	1
STAU1	0.010893	9.23E-06	MSR Acceptor	1	0	0	0

Only annotated introns overlapping all age groups and tissues were used, corresponding to N=139,419 introns. Inaccurate splicing at the 5'ss was evaluated through the "MSR Donor" measure. Inaccurate splicing at the 3'ss was evaluated through the "MSR Acceptor" measure. Focusing on the 'MSR Donor' measure: "H0 = The introns in the '20-39' & '60-79' sample groups present distributions of 'MSR_D' values that are symmetric about their median value after accounting for the fold-change in TPM expression with age of each RBP/NMD factor evaluated". "H1 = The introns in the '60-79' sample group present a distribution of higher 'MSR_Donor' values compared to the 20-39 yrs group, indicating a higher frequency of inaccurate splicing activity after accounting for the fold-change in TPM expression with age of each RBP/NMD factor evaluated". We repeated these methods to test for MSR_Acceptor differences. p-values were adjusted using the False Discovery Rate (FDR) method. Non-significant FDR values (i.e. the alternative hypothesis is rejected) indicate that, after normalising mis-splicing levels by individual changes in expression levels with age of each RBP/NMD gene studied, increases in splicing noise previously observed with age are not detectable.

Supplementary Table 9. Ranked top 10 outcomes (ordered by ascending effect size result) corresponding from the one-tailed, paired Wilcoxon effect size test to evaluate the probability of superior normalised MSR values with increasing age across introns from blood vessel tissue samples.

RBP/NMD factor	Effect Size	FDR (corrected p values)	MSR Type	Splicing Regulation	Spliceosome	Exon Junction Complex	NMD
DDX5	0.001753	1	MSR Donor	1	0	0	0
PSIP1	0.001846	1	MSR Donor	1	0	0	0
PTBP3	0.002017	1	MSR Donor	1	0	0	0
PCBP2	0.002206	1	MSR Acceptor	1	0	0	0
TRA2A	0.002561	0.857926	MSR Acceptor	1	0	0	0
SMNDC1	0.002858	1	MSR Donor	0	1	0	0
RAVER1	0.003149	1	MSR Donor	1	0	0	0
CCAR2	0.003592	1	MSR Donor	1	1	0	0
KHDRBS3	0.007298	0.076491	MSR Acceptor	1	0	0	0
CELF1	0.007919	1	MSR Donor	1	0	0	0

Only annotated introns overlapping all age groups and tissues were used, corresponding to N=139,419. Inaccurate splicing at the 5’ss was evaluated through the “MSR Donor” measure. Inaccurate splicing at the 3’ss was evaluated through the “MSR Acceptor” measure. Focusing on the ‘MSR Donor’ measure: “H0 = The introns in the '20-39' & '60-79' sample groups present distributions of ‘MSR_D’ values that are symmetric about their median value after accounting for the fold-change in TPM expression with age of each RBP/NMD factor evaluated”. “H1 = The introns in the '60-79' sample group present a distribution of higher ‘MSR_Donor’ values compared to the 20-39 yrs group, indicating a higher frequency of inaccurate splicing activity after accounting for the fold-change in TPM expression with age of each RBP/NMD factor evaluated”. We repeated these methods to test for MSR_Acceptor differences. p-values were adjusted using the False Discovery Rate (FDR) method. Non-significant FDR values (i.e. the alternative hypothesis is rejected) indicate that, after normalising mis-splicing levels by individual changes in expression levels with age of each RBP/NMD gene studied, increases in splicing noise previously observed with age are not detectable.

Supplementary Table 10. Ranked top 10 outcomes (ordered by ascending effect size result) corresponding from the one-tailed and paired Wilcoxon effect size test to evaluate the probability of superior normalised MSR values with increasing age across introns from brain tissue samples.

RBP/NMD factor	Effect Size	FDR (corrected p values)	MSR Type	Splicing Regulation	Spliceosome	Exon Junction Complex	NMD
NCBP2	0.002153	1	MSR Acceptor	1	0	0	0
PSIP1	0.002799	1	MSR Donor	1	0	0	0
DDX42	0.004203	1	MSR Donor	0	1	0	0
GPKOW	0.004475	1	MSR Donor	1	1	0	0
QKI	0.005361	1	MSR Donor	1	0	0	0
ZRANB2	0.006158	1	MSR Donor	1	0	0	0
RBM25	0.006271	1	MSR Donor	1	1	0	0
AGO2	0.006459	1	MSR Acceptor	1	0	0	0
HNRNPK	0.006664	1	MSR Acceptor	1	0	0	0
RBM25	0.009528	1	MSR Acceptor	1	1	0	0

Only annotated introns overlapping all age groups and tissues were used, corresponding to N=139,419. Inaccurate splicing at the 5’ss was evaluated through the “MSR Donor” measure. Inaccurate splicing at the 3’ss was evaluated through the “MSR Acceptor” measure. Focusing on the ‘MSR Donor’ measure: “H0 = The introns in the '20-39' & '60-79' sample groups present distributions of ‘MSR_D’ values that are symmetric about their median value after accounting for the fold-change in TPM expression with age of each RBP/NMD factor evaluated”. “H1 = The introns in the '60-79' sample group present a distribution of higher ‘MSR_Donor’ values compared to the 20-39 yrs group, indicating a higher frequency of inaccurate splicing activity after accounting for the fold-change in TPM expression with age of each RBP/NMD factor evaluated”. We repeated these methods to test for MSR_Acceptor differences. p-values were adjusted using the False Discovery Rate (FDR) method. Non-significant FDR values (i.e. the alternative hypothesis is rejected) indicate that, after normalising mis-splicing levels by individual changes in expression levels with age of each RBP/NMD gene studied, increases in splicing noise previously observed with age are not detectable.

Supplementary Table 11. Expression Weighted Cell Type Enrichment analysis to determine whether RBP factors involved in splicing regulation have higher expression levels within particular brain-related cell types than would be expected by chance.

Cell Type	p-value	Fold change	SD from mean	FDR (corrected p values)
GABAergic_LAMP5	0.0129	1.53705	2.921257	0.084433
Glutamatergic_L4_IT	0.0214	1.471325	2.481209	0.084433
GABAergic_PAX6	0.0255	1.497309	2.394422	0.084433
Glutamatergic_IT	0.0266	1.376028	2.244713	0.084433
GABAergic_SST	0.0289	1.444402	2.321911	0.084433
Glutamatergic_L5_6_IT_Car3	0.0298	1.450241	2.22228	0.084433
GABAergic_PVALB	0.0364	1.366914	2.015943	0.0884
Glutamatergic_L5_6_NP	0.0451	1.41605	1.989512	0.095389
Glutamatergic_L6b	0.0505	1.354495	1.783631	0.095389
Glutamatergic_L6_CT	0.0648	1.298674	1.580784	0.11016
GABAergic_VIP	0.0778	1.338982	1.564835	0.120236
OPC	0.1205	1.295944	1.199027	0.170708
Glutamatergic_L5_ET	0.1604	1.20857	0.877792	0.195257
Oligodendrocyte	0.1608	1.27168	0.960102	0.195257
Microglia	0.2543	1.182894	0.585017	0.288207
Astrocyte	0.3106	1.098481	0.368508	0.330013
Vascular_cells	0.3405	1.10236	0.316492	0.3405

Level 1 cell-type annotation. p-values were adjusted using the False Discovery Rate (FDR) method. The statistical test used corresponded to the mean specificity score of the hit genes for each cell type (EWCE::bootstrap_enrichment_test function, one-sided test, EWCE R package, version 1.4.0).

Supplementary Table 12. Expression Weighted Cell Type Enrichment analysis to determine whether RBP factors involved in spliceosomal function have higher expression levels within particular brain-related cell types than would be expected by chance.

Cell Type	p-value	Fold change	SD from mean	FDR (corrected p values)
GABAergic_LAMP5	0.0011	1.375122	3.755623	0.010767
Glutamatergic_L4_IT	0.0013	1.402893	3.707585	0.010767
GABAergic_PAX6	0.0019	1.431195	3.756322	0.010767
Glutamatergic_IT	0.0046	1.384674	3.068386	0.01955
GABAergic_SST	0.0069	1.363469	2.959474	0.02346
Glutamatergic_L5_6_IT_Car3	0.0087	1.330729	2.952198	0.02465
GABAergic_PVALB	0.0105	1.278749	2.654812	0.0255
Glutamatergic_L5_6_NP	0.0143	1.27615	2.492228	0.030388
Glutamatergic_L6b	0.017	1.346065	2.387958	0.032111
Glutamatergic_L6_CT	0.0299	1.289447	2.167283	0.05083
GABAergic_VIP	0.0372	1.364151	1.934846	0.057491
OPC	0.0479	1.228278	1.827242	0.061564
Glutamatergic_L5_ET	0.0505	1.222403	1.798673	0.061564
Oligodendrocyte	0.0507	1.296633	1.761784	0.061564
Microglia	0.1444	1.209687	1.058157	0.163653
Astrocyte	0.2117	1.106901	0.741488	0.224931
Vascular_cells	0.2252	1.11245	0.70826	0.2252

Level 1 cell-type annotation. p-values were adjusted using the False Discovery Rate (FDR) method. The statistical test used corresponded to the mean specificity score of the hit genes for each cell type (EWCE::bootstrap_enrichment_test function, one-sided test, EWCE R package, version 1.4.0).

Supplementary Table 13. Ranked top 10 outcomes (ordered by ascending effect size result) corresponding from the one-tailed, paired Wilcoxon effect size test to evaluate the probability of superior normalised MSR values in disease status (AD) compared to unaffected control samples.

RBP/NMD factor	Effect Size	FDR	MSR Type	Splicing Regulation	Spliceosome	Exon Junction Complex	NMD
NCBP2	0.002153	1	MSR Acceptor	1	0	0	0
PSIP1	0.002799	1	MSR Donor	1	0	0	0
DDX42	0.004203	1	MSR Donor	0	1	0	0
GPKOW	0.004475	1	MSR Donor	1	1	0	0
QKI	0.005361	1	MSR Donor	1	0	0	0
ZRANB2	0.006158	1	MSR Donor	1	0	0	0
RBM25	0.006271	1	MSR Donor	1	1	0	0
AGO2	0.006459	1	MSR Acceptor	1	0	0	0
HNRNPK	0.006664	1	MSR Acceptor	1	0	0	0
RBM25	0.009528	1	MSR Acceptor	1	1	0	0

Only annotated introns overlapping AD and control sample groups were used, corresponding to N=203,411. Inaccurate splicing at the 5’ss was evaluated through the “MSR Donor” measure. Inaccurate splicing at the 3’ss was evaluated through the “MSR Acceptor” measure. Focusing on the ‘MSR Donor’ measure: “H0 = The introns in the ‘AD’ and ‘control’ sample groups present symmetric distributions of ‘MSR_D’ values around their median value after accounting for the fold-change in TPM expression levels of each RBP/NMD factor evaluated between the two sample groups”. “H1 = The introns in the ‘AD’ sample group present a distribution of higher ‘MSR_Donor’ values compared to the ‘control’ group, indicating a higher frequency of inaccurate splicing activity after accounting for the fold-change in TPM expression levels of each RBP/NMD factor evaluated between the two sample groups”. We repeated these methods to test for MSR_Acceptor differences. p-values were adjusted using the False Discovery Rate (FDR) method. Non-significant FDR values (i.e.the alternative hypothesis is rejected) indicate that, after normalising mis-splicing levels by individual changes in expression of each RBP/NMD gene studied between the control and disease group, differences in splicing noise between AD and control sample groups are not detectable. AD/control dataset corresponded to GEO:GSE95587.

Supplementary Table 14. List of GTEx body sites and tissues as classified by the recount3 project.

**Recount3 Project ID**	**Body Site**	**Tissue**
ADIPOSE_TISSUE	ADIPOSE TISSUE	Adipose - Subcutaneous
		Adipose - Visceral (Omentum)
ADRENAL_GLAND	ADRENAL GLAND	Adrenal Gland
BLOOD	BLOOD	Cells - EBV-transformed lymphocytes
		Whole Blood
BLOOD_VESSEL	BLOOD VESSEL	Artery - Aorta
		Artery - Coronary
		Artery - Tibial
BRAIN	BRAIN	Brain - Amygdala
		Brain - Anterior cingulate cortex (BA24)
		Brain - Caudate (basal ganglia)
		Brain - Cerebellar Hemisphere
		Brain - Frontal Cortex (BA9)
		Brain - Hippocampus
		Brain - Hypothalamus
		Brain - Nucleus accumbens (basal ganglia)
		Brain - Putamen (basal ganglia)
		Brain - Spinal cord (cervical c-1)
		Brain - Substantia nigra
COLON	COLON	Colon - Sigmoid
		Colon - Transverse
ESOPHAGUS	ESOPHAGUS	Esophagus - Gastroesophageal Junction
		Esophagus - Mucosa
		Esophagus - Muscularis
HEART	HEART	Heart - Atrial Appendage
		Heart - Left Ventricle
KIDNEY	KIDNEY	Kidney - Cortex
		Kidney - Medulla
LIVER	LIVER	Liver
LUNG	LUNG	Lung
MUSCLE	MUSCLE	Muscle - Skeletal
NERVE	NERVE	Nerve - Tibial
PANCREAS	PANCREAS	Pancreas
PITUITARY	PITUITARY	Pituitary
SALIVARY_GLAND	SALIVARY GLAND	Minor Salivary Gland
SKIN	SKIN	Cells - Cultured fibroblasts
		Skin - Not Sun Exposed (Suprapubic)
		Skin - Sun Exposed (Lower leg)
SMALL_INTESTINE	SMALL INTESTINE	Small Intestine - Terminal Ileum
SPLEEN	SPLEEN	Spleen
STOMACH	STOMACH	Stomach
THYROID	THYROID	Thyroid
