# Supplementary material for: Splicing accuracy varies across human introns, tissues, age and disease
Source: Nat Commun. 2025 Jan 27;16:1068. doi: 10.1038/s41467-024-55607-x (PMC11772838; doi:10.1038/s41467-024-55607-x)
Supplement: Supplementary file 2 — Reporting Summary [file 41467_2024_55607_MOESM2_ESM.pdf]

Corresponding author(s): Prof. Mina Ryten

Last updated by author(s): Dec 4, 2024

Reporting Summary

Nature Portfolio wishes to improve the reproducibility of the work that we publish. This form provides structure for consistency and transparency in reporting. For further information on Nature Portfolio policies, see our Editorial Policies and the Editorial Policy Checklist.

Statistics

For all statistical analyses, confirm that the following items are present in the figure legend, table legend, main text, or Methods section.

n/a	Confirmed	
☐	☒	The exact sample size (*n*) for each experimental group/condition, given as a discrete number and unit of measurement
☐	☒	A statement on whether measurements were taken from distinct samples or whether the same sample was measured repeatedly
☐	☒	The statistical test(s) used AND whether they are one- or two-sided<br>*Only common tests should be described solely by name; describe more complex techniques in the Methods section.*
☐	☒	A description of all covariates tested
☐	☒	A description of any assumptions or corrections, such as tests of normality and adjustment for multiple comparisons
☐	☒	A full description of the statistical parameters including central tendency (e.g. means) or other basic estimates (e.g. regression coefficient) AND variation (e.g. standard deviation) or associated estimates of uncertainty (e.g. confidence intervals)
☐	☒	For null hypothesis testing, the test statistic (e.g. *F*, *t*, *r*) with confidence intervals, effect sizes, degrees of freedom and *P* value noted<br>*Give P values as exact values whenever suitable.*
☒	☐	For Bayesian analysis, information on the choice of priors and Markov chain Monte Carlo settings
☒	☐	For hierarchical and complex designs, identification of the appropriate level for tests and full reporting of outcomes
☐	☒	Estimates of effect sizes (e.g. Cohen's *d*, Pearson's *r*), indicating how they were calculated

Our web collection on statistics for biologists contains articles on many of the points above.

Software and code

Policy information about availability of computer code

Data collection	This manuscript processes publicly available RNA-sequencing data that is already in the public domain. To download each of the RNA-seq datasets studied, we used the recount3 R package (version 1.0.7, https://github.com/LieberInstitute/recount3), to download 1) data corresponding to the GTEx v8 project (Supplementary Table 14); and 2) projectID = "SRP100948" (Gene Expression Omnibus: GSE95587). Bam files from the ENCODE Gene Silencing Series were downloaded using the R code https://github.com/SoniaRuiz/recount3-database-project/ (https://doi.org/10.5281/zenodo.14204939, v2.0.0, R script 29_ENCODE_download_bams.R, which was adapted from (https://github.com/guillermo1996/ENCODE_Metadata_Extraction, version 1.0.2, DOI: 10.5281/zenodo.7733986).
Data analysis	Code to build the databases can be found:https://github.com/SoniaRuiz/recount3-database-project (version 2.0.0, DOI: 10.5281/zenodo.14204939)<br>Code to produce the results indicated in the manuscript: https://github.com/SoniaRuiz/splicing-accuracy-manuscript (version 2.0.0, DOI: 10.5281/zenodo.14204490).<br>All analyses were performed in R version 4.0.2 (Ubuntu 16.04.7 LTS).<br>- Regtools software package (version 0.5.2, http://regtools.org/).<br>- SAMTOOLS software (version 1.16.1, RRID:SCR_002105, http://htslib.org/).<br>- R package rstatix version 0.7.1, RRID:SCR_021240, https://CRAN.R-project.org/package=rstatix.<br>- wilcox_effsize() (R package rstatix, version 0.7.1, RRID:SCR_021240, https://CRAN.R-project.org/package=rstatix).<br>- PyMuPDF (version 1.21.1, https://github.com/pymupdf/PyMuPDF).<br>- pytesseract (version 0.3.10, https://pypi.org/project/pytesseract/)<br>- chi square test (function chisq.test, R package stats, version 4.0.5).<br>- create_rse_manual() (R package recount3, version 1.0.7, https://bioconductor.org/packages/release/bioc/html/recount3.html).<br>- transform_counts() (R package recount3, version 1.0.7, https://bioconductor.org/packages/release/bioc/html/recount3.html)

- enrichGO (R package clusterProfiler, version 3.18.1, RRID:SCR_016884, http://yulab-smu.top/biomedical-knowledge-mining-book/clusterprofiler-go.html).
- enrichKEGG (R package clusterProfiler, version 3.18.1, RRID:SCR_016884, http://yulab-smu.top/biomedical-knowledge-mining-book/clusterprofiler-kegg.html?q=enrichKEGG#clusterprofiler-kegg-pathway-ora).
- EWCE R package (version 0.99.3, https://bioconductor.org/packages/release/bioc/html/EWCE.html).
- dasper version 1.4.3 (https://bioconductor.org/packages/release/bioc/html/dasper.html).

For manuscripts utilizing custom algorithms or software that are central to the research but not yet described in published literature, software must be made available to editors and reviewers. We strongly encourage code deposition in a community repository (e.g. GitHub). See the Nature Portfolio guidelines for submitting code & software for further information.

Data

Policy information about availability of data

All manuscripts must include a data availability statement. This statement should provide the following information, where applicable:

- Accession codes, unique identifiers, or web links for publicly available datasets
- A description of any restrictions on data availability
- For clinical datasets or third party data, please ensure that the statement adheres to our policy

The repositories https://github.com/SoniaRuiz/recount3-database-project (version 2.0.0, DOI: https://doi.org/10.5281/zenodo.14204939) and https://github.com/SoniaRuiz/splicing-accuracy-manuscript (version 2.0.0, https://doi.org/10.5281/zenodo.14204490) contain the code (1) to generate the five sqlite databases described in this manuscript and (2) to replicate all analyses, figures, tables and supplementary information included in this manuscript, respectively. All analyses were performed in R version 4.0.2 (Ubuntu 16.04.7 LTS).

The code used to obtain the metadata and extract the bam files associated with each ENCODE shRNA knockdown data was adapted from https://github.com/guillermo1996/ENCODE_Metadata_Extraction (version 1.0.2, DOI: 10.5281/zenodo.7733986) and https://github.com/guillermo1996/ENCODE_Splicing_Analysis (version 1.0.1, DOI: 10.5281/zenodo.7733984).

The code to calculate the expression levels of the RBPs known to contribute to splicing and its regulation across body sites can be accessed at https://github.com/ainefairbrother/RBP_expression_analysis (version 1.0.0, DOI: 10.5281/zenodo.7736907).

The code to reproduce the cell type specificity analysis of the set of RBPs known to contribute to splicing and its regulation, and using as reference the drop-seq data from multiple cortical regions (Allen Brain Atlas) is available at: https://github.com/mgrantpeters/RBP_EWCE_analysis (version 1.0, DOI: 10.5281/zenodo.7734035).

The code to generate the cell type dataset using the function generate_celltype_data() from the R package EWCE, can be accessed at: https://github.com/RHReynolds/MarkerGenes (version 0.99.1, DOI: 10.5281/zenodo.6418604).

Research involving human participants, their data, or biological material

Policy information about studies with human participants or human data. See also policy information about sex, gender (identity/presentation), and sexual orientation and race, ethnicity and racism.

Reporting on sex and gender	No analyses conducted were stratified on sex or gender. Self reported biological sex was used as a covariate.
Reporting on race, ethnicity, or other socially relevant groupings	GTEx v8 project: Although both sexes were enrolled in the study, males exceeded females with a ratio of 2:1. Furthermore, of the 948 individuals included in the V8 release, only 123 were Black or African American individuals, Asian, American Indian or Alaskan Native. The remaining 831 individuals either had no reported ethnicity (6 individuals) or were reported to be white.<br><br>GSE95587: no ethnicity information was provided; metadata available: pathology/clinical reports, including age, sex, diagnosis, and Braak stage.<br><br>ENCODE Gene Silencing Series: shRNA protein knockdown data from Homo sapiens in K562 (female, 53 years old) and HepG2 cells lines (male, 15 years old) was downloaded.
Population characteristics	GTEx v8: junction data on 17,510 samples across 948 post-mortem donors and 54 tissues after accounting for the exclusion of samples that did not meet the minimum GTEx quality control criteria (gtex.smafrze = 'EXCLUDE'; n=1,571).<br><br>ENCODE gene-silencing series: We downloaded 432 .bam files corresponding to two donors: K562 cell lines were collected from a female (adult, 53 years-old), whereas HepG2 cell lines were obtained from a male (child, 15 years-old).<br><br>Gene Expression Omnibus: GSE95587. We downloaded from recount3 (recount3 project ID = "SRP100948") junction data corresponding to 117 fusiform gyrus samples originating from individuals with Alzheimer's (AD) and neurologically normal (control) individuals; Friedman et. al. (2018).
Recruitment	Please see (GTEx Consortium Science. 2020) and (Friedman et. al. 2018).
Ethics oversight	Ethics evaluation plan was not required in this study. All data sources (GTEx, ENCODE gene-silencing series and GEO:GSE95587) had ethical approval.

Note that full information on the approval of the study protocol must also be provided in the manuscript.

Field-specific reporting

Please select the one below that is the best fit for your research. If you are not sure, read the appropriate sections before making your selection.

☒ Life sciences ☐ Behavioural & social sciences ☐ Ecological, evolutionary & environmental sciences

For a reference copy of the document with all sections, see nature.com/documents/nr-reporting-summary-flat.pdf

Life sciences study design

All studies must disclose on these points even when the disclosure is negative.

Sample size	After quality-control, we studied and processed:<br>- 13,949 samples from the GTEx v8 project and 6,519 GTEx v8 samples in the age stratification analysis.<br>- 432 bam sample files from the ENCODE gene-silencing series,<br>- 48 samples from the 'GEO: GSE95587' dataset.
Data exclusions	GTEx v8: we discarded samples with RIN < 6, samples not fresh frozen preserved, samples from sex-specific tissues and from tissues with less than 70 samples. We discarded samples with metadata "gtex.smafrze = 'EXCLUDE'".<br><br>ENCODE: we only downloaded gene-silencing series experiment with 8 experiments with RNA-seq data available on shRNA knockdown (4 samples) and control (4 samples) in K562 and HepG2 cells.<br><br>GSE95587: we only analysed 24 AD-diagnosed samples and 24 unaffected samples (controls) with matching mapped read depth (matchIt function, R package rstatix version 0.7.1, RRID:SCR_021240, https://CRAN.R-project.org/package=rstatix.).
Replication	Results obtained using the GTEx dataset were robustly replicated even after i) increasing the minimum number of supporting split reads required for a junction to be considered (from 1 to 2 split reads), and ii) after increasing the stringency of read alignment by raising the anchor length required (from 5 bp to 8 bp).<br>Results were also replicated using the independent RNA-seq dataset GEO:GSE95587.
Randomization	Randomisation was not applicable to this study as no data was analysed that would allow identification of any individuals in the study.
Blinding	n/a

Behavioural & social sciences study design

All studies must disclose on these points even when the disclosure is negative.

Study description	n/a
Research sample	n/a
Sampling strategy	n/a
Data collection	n/a
Timing	n/a
Data exclusions	n/a
Non-participation	n/a
Randomization	n/a

Ecological, evolutionary & environmental sciences study design

All studies must disclose on these points even when the disclosure is negative.

Study description	n/a
Research sample	n/a
Sampling strategy	n/a
Data collection	n/a

Timing and spatial scale	n/a
Data exclusions	n/a
Reproducibility	n/a
Randomization	n/a
Blinding	n/a

Did the study involve field work? ☐ Yes ☒ No

Field work, collection and transport

Field conditions	n/a
Location	n/a
Access & import/export	n/a
Disturbance	n/a

Reporting for specific materials, systems and methods

We require information from authors about some types of materials, experimental systems and methods used in many studies. Here, indicate whether each material, system or method listed is relevant to your study. If you are not sure if a list item applies to your research, read the appropriate section before selecting a response.

Materials & experimental systems

n/a	Involved in the study	
☒	☐	Antibodies
☒	☐	Eukaryotic cell lines
☒	☐	Palaeontology and archaeology
☒	☐	Animals and other organisms
☒	☐	Clinical data
☒	☐	Dual use research of concern
☒	☐	Plants

Methods

n/a	Involved in the study	
☒	☐	ChIP-seq
☒	☐	Flow cytometry
☒	☐	MRI-based neuroimaging

Antibodies

Antibodies used	n/a
Validation	n/a

Eukaryotic cell lines

Policy information about cell lines and Sex and Gender in Research

Cell line source(s)	n/a
Authentication	n/a
Mycoplasma contamination	n/a
Commonly misidentified lines (See ICLAC register)	n/a

Palaeontology and Archaeology

Specimen provenance	n/a
Specimen deposition	n/a
Dating methods	n/a

☐ Tick this box to confirm that the raw and calibrated dates are available in the paper or in Supplementary Information.

Ethics oversight	n/a

Note that full information on the approval of the study protocol must also be provided in the manuscript.

Animals and other research organisms

Policy information about studies involving animals; ARRIVE guidelines recommended for reporting animal research, and Sex and Gender in Research

Laboratory animals	n/a
Wild animals	n/a
Reporting on sex	n/a
Field-collected samples	n/a
Ethics oversight	n/a

Note that full information on the approval of the study protocol must also be provided in the manuscript.

Clinical data

Policy information about clinical studies

All manuscripts should comply with the ICMJE guidelines for publication of clinical research and a completed CONSORT checklist must be included with all submissions.

Clinical trial registration	n/a
Study protocol	n/a
Data collection	n/a
Outcomes	n/a

Dual use research of concern

Policy information about dual use research of concern

Hazards

Could the accidental, deliberate or reckless misuse of agents or technologies generated in the work, or the application of information presented in the manuscript, pose a threat to:

No	Yes	
☒	☐	Public health
☒	☐	National security
☒	☐	Crops and/or livestock
☒	☐	Ecosystems
☒	☐	Any other significant area

Experiments of concern

Does the work involve any of these experiments of concern:

No	Yes	
☒	☐	Demonstrate how to render a vaccine ineffective
☒	☐	Confer resistance to therapeutically useful antibiotics or antiviral agents
☒	☐	Enhance the virulence of a pathogen or render a nonpathogen virulent
☒	☐	Increase transmissibility of a pathogen
☒	☐	Alter the host range of a pathogen
☒	☐	Enable evasion of diagnostic/detection modalities
☒	☐	Enable the weaponization of a biological agent or toxin
☒	☐	Any other potentially harmful combination of experiments and agents

Plants

Seed stocks	n/a
Novel plant genotypes	n/a
Authentication	n/a

ChIP-seq

Data deposition

☐ Confirm that both raw and final processed data have been deposited in a public database such as GEO.

☐ Confirm that you have deposited or provided access to graph files (e.g. BED files) for the called peaks.

Data access links<br>*May remain private before publication.*	n/a
Files in database submission	n/a
Genome browser session<br>(e.g. UCSC)	n/a

Methodology

Replicates	n/a
Sequencing depth	n/a
Antibodies	n/a
Peak calling parameters	n/a
Data quality	n/a
Software	n/a

Flow Cytometry

Plots

Confirm that:

☐ The axis labels state the marker and fluorochrome used (e.g. CD4-FITC).

☐ The axis scales are clearly visible. Include numbers along axes only for bottom left plot of group (a 'group' is an analysis of identical markers).

☐ All plots are contour plots with outliers or pseudocolor plots.

☐ A numerical value for number of cells or percentage (with statistics) is provided.

Methodology

Sample preparation	n/a
Instrument	n/a
Software	n/a
Cell population abundance	n/a
Gating strategy	n/a

☐ Tick this box to confirm that a figure exemplifying the gating strategy is provided in the Supplementary Information.

Magnetic resonance imaging

Experimental design

Design type	n/a
Design specifications	n/a
Behavioral performance measures	n/a

Acquisition

Imaging type(s)	n/a
Field strength	n/a
Sequence & imaging parameters	n/a
Area of acquisition	n/a

Diffusion MRI ☐ Used ☒ Not used

Preprocessing

Preprocessing software	n/a
Normalization	n/a
Normalization template	n/a
Noise and artifact removal	n/a
Volume censoring	n/a

Statistical modeling & inference

Model type and settings	n/a
Effect(s) tested	n/a

Specify type of analysis: ☐ Whole brain ☐ ROI-based ☐ Both

Statistic type for inference<br>(See Eklund et al. 2016)	n/a
Correction	n/a

Models & analysis

n/a	Involved in the study
☒	☐ Functional and/or effective connectivity
☒	☐ Graph analysis
☒	☐ Multivariate modeling or predictive analysis

Functional and/or effective connectivity	n/a
Graph analysis	n/a
Multivariate modeling and predictive analysis	n/a
